# Insights on the structure–function relationship of human multidrug resistance protein 7 (MRP7/ABCC10) from molecular dynamics simulations and docking studies

**DOI:** 10.1002/mco2.65

**Published:** 2021-03-25

**Authors:** Jing‐Quan Wang, Qingbin Cui, Zi‐Ning Lei, Qiu‐Xu Teng, Ning Ji, Lusheng Lin, Zhijun Liu, Zhe‐Sheng Chen

**Affiliations:** ^1^ Department of Pharmaceutical Sciences College of Pharmacy and Health Sciences St. John's University Queens New York USA; ^2^ School of Public Health Guangzhou Medical University Guangzhou China; ^3^ Cell Research Center Shenzhen Bolun Institute of Biotechnology Shenzhen China; ^4^ Department of Medical Microbiology Weifang Medical University Weifang China

**Keywords:** ABC transporters, ABCC10, homology modeling, molecular dynamics, MRP7

## Abstract

ATP‐binding cassette (ABC) transporters superfamily mediates multidrug resistance in cancer by extruding structurally distinct chemotherapeutic agents, causing failure in chemotherapy. Among the 49 ABC transporters, multidrug resistance protein 7 (MRP7 or ABCC10) is relatively new and has been identified as the efflux pump of multiple anticancer agents including *Vinca* alkaloids and taxanes. Herein, we construct and validate a homology model for human MRP7 based on the cryo‐EM structures of MRP1. Structure–function relationship of MRP7 was obtained from molecular dynamics simulations and docking studies and was in accordance with previous studies of ABC transporters. The motion patterns correlated with efflux mechanism were discussed. Additionally, predicted substrate‐ and modulator‐binding sites of MRP7 were described for the first time, which provided rational insights in understanding the drug binding and functional regulation in MRP7. Our findings will benefit the high‐throughput virtual screening and development of MRP7 modulators in the future.

## Graphical abstract

Homology modeling of MRP7 and predicted binding pocket characterization via docking analysis.

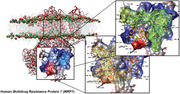

## 1 INTRODUCTION

Adenosine triphosphate (ATP) ‐binding cassette (ABC) transporters are known for mediating chemoresistance through the efflux of anticancer agents. They belong to a superfamily of ATP‐powered active membrane transporters that widely expressed in various tissues.^1^ The ABC transporter superfamily is composed of seven subfamilies named ABCA to ABCG based on their structural and functional features.^2^, ^3^ Besides transporting chemotherapeutic agents in some cancer cells, ABC transporters are also responsible for the transportation of a broad spectrum of endobiotics such as lipids as well as metabolic products across membranes.^4^

Till now, 12 ABCC transporters have been discovered, and there are nine members (ABCC1‐6 and ABCC10‐12) also referred as “multidrug resistance protein (MRP)” due to their ability to confer multidrug resistance (MDR) in cancers.^3^ The ABCC family members could be divided structurally into “long” (ABCC1, ABCC2, ABCC3, ABCC6, and ABCC10) and “short” (ABCC4, ABCC5, ABCC11, and ABCC12). Both classes share common structures of a typical ABC transporter with two transmembrane domains (TMDs) and two nucleotide‐binding domains (NBDs). “Long” class members share a third transmembrane domain (TMD0) with five helices of which the function is still unclear.^4^, ^5^

ABCC10/MRP7 was first discovered in 2001 from expressed sequence tag databases mining^6^ and its transport properties were determined subsequently.^7^ MRP7 was proved to be able to transport multiple types of substrates including amphipathic anions such as 17β‐estradiol 17‐(β‐D‐glucuronide) (E_2_17βG), natural product and derivatives including *vinca* alkaloids such as vincristine, and taxanes such as paclitaxel.^6^, ^8^ A study of expression profiles of transporter genes in human tissues revealed that MRP7 is widely expressed in different tissues including brain, kidney, liver, pancreas, stomach, colon, intestine, and lung.^9^ Other ABC transporters such as P‐gp, BCRP, or MRP1 have been demonstrated expressing in tissues with secretory or excretory functions (liver, kidney, and gastrointestinal tract) and at blood–brain barrier.^10^, ^11^, ^12^ MRP7 also showed similar expression pattern thus suggesting its involvement in transporting endogenous molecules. In vitro and in vivo studies have suggested that MRP7 was responsible for mediating MDR in cancer cells,^13^, ^14^, ^15^ and downregulated MRP7 expression by targeting its gene expression could enhance cellular sensitivity to chemotherapeutic drugs.^16^ Besides, our group has discovered that MRP7 could be functionally regulated by tyrosine kinase inhibitors, phosphodiesterase inhibitors, Raf kinase inhibitors, fibroblast growth factor inhibitors, and other small molecule drugs, leading to reversed MDR in resistant cancer cells.^17^, ^18^, ^19^, ^20^, ^21^, ^22^

Clinically, MRP7 has been reported to play important roles in acquired MDR and the prognosis of certain cancers.^9^, ^23^ Additionally, MRP7 participates in FOXM‐induced 5‐FU resistance in colorectal cancer patients based on the strong correlation between mRNA levels of MRP7 and FOXM in tumor tissues. Furthermore, MRP7 also contributes to alteration in intracellular permeation of nevirapine, a nonnucleoside reverse transcriptase inhibitor for HIV‐1.^24^ As a result, further understanding the structural features and transportation mechanisms of MRP7 is crucial in increasing the survival rate of patients with limited response to chemotherapy due to acquired MDR, as well as developing and discovering substrates/modulators to overcome MRP7‐mediated MDR or decreasing unexpected synergistic toxicity in combinational chemotherapies. However, due to the difficulty and cost in obtaining crystal structure of membrane protein, no high‐resolution structure of human MRP7 is available so far. Previously, the cryo‐EM structure of bovine MRP1 was reported at 3.1–3.5 Å with inward‐ and outward‐facing conformations.^25^, ^26^ Although there are significant differences between MRP1 and MRP7 in length, size, amino acid sequence, and transportation pattern, it still provides possibility to construct high‐quality homology models of MRP7 via computational strategies. Here, we present the homology models of MRP7 combining current knowledge of the transporter and the homology modeling methods based on the cryo‐EM structure of MRP1 in order to (1) provide a functionally validated human MRP7 homology model; (2) assess the structural dynamics to identify potential conformational changes associated with efflux mechanism; and (3) evaluate the behavior of reported substrates and modulators of MRP7 by analyzing ligand–protein interactions. Also, extra docking simulations were performed using template MRP1 and our homology model to further validate that the MRP7 model was not biased toward its templates. Our study will provide rational insights in the drug development or repurposing of MRP7 modulators.

## 2 RESULTS

### 2.1 MRP7 homology modeling and structure refinement

A proper template is determined by multiple factors including sequence identity, resolution, functional similarity, and sequence alignment, which are essential for identifying conserved regions, ligand‐binding sites as well as structural domains.^27^ Here, the bovine MRP1 (MRP1) with inward‐facing (PDB ID: 5UJA)^25^ conformations was chosen as modeling template according to the high identity rate among top alignments shown in Table S1. The resolution of 5UJA is 3.34 Å.

**FIGURE 1:**
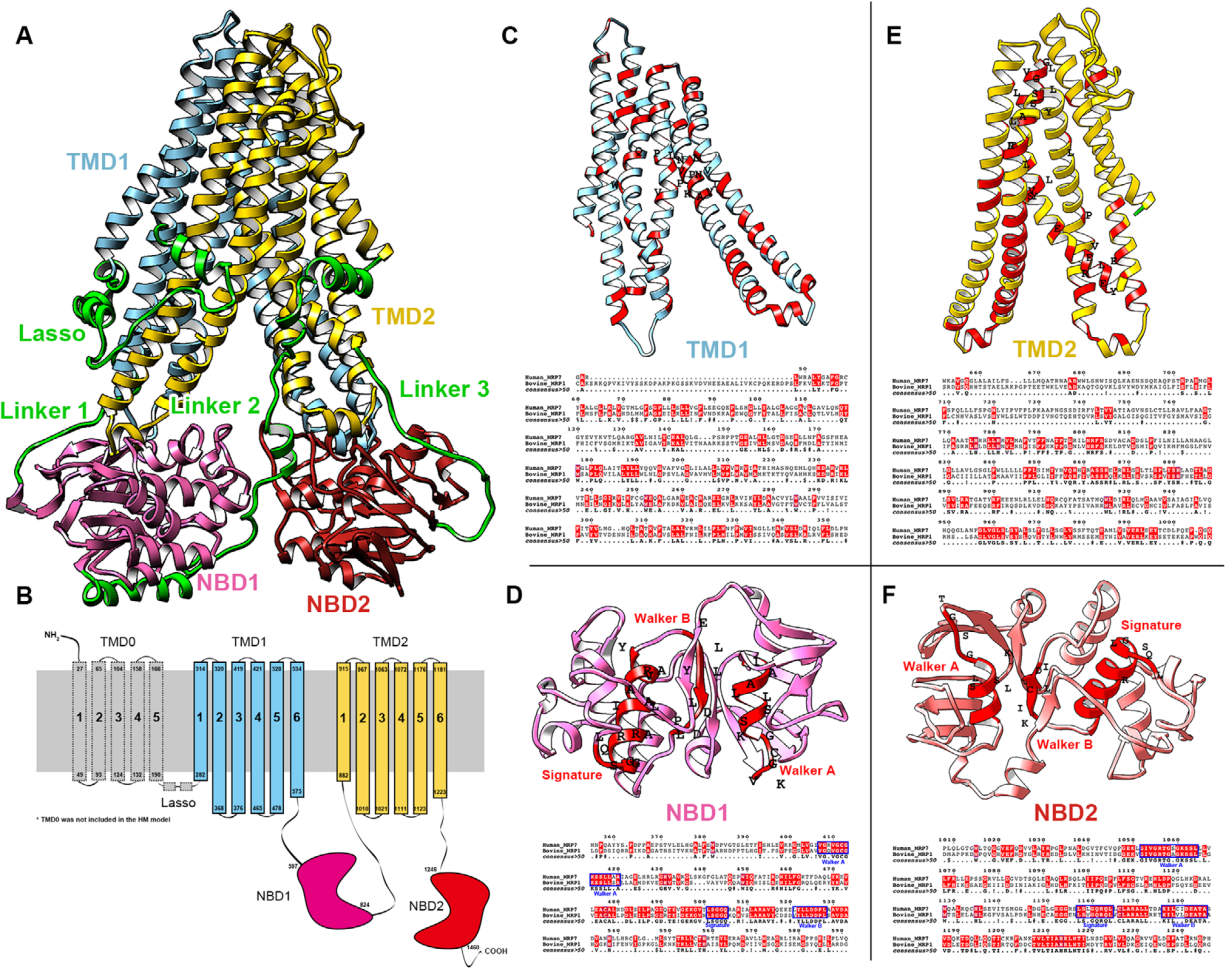
Sequence alignment of MRP1 and MRP7. (A) Homology model of human MRP7. Key domains were colored as Lasso/Linker 1/Linker 2/Linker 3: green; TMD1: blue; TMD2: yellow; NBD1: pink; BND2: red. (B) Topological structure of human MRP7. Key domains were colored and labeled. (C–F) Top: Predicted TMD1 (C) / NBD1 (D) / TMD2 (E) / NBD2 (F) structures of human MRP7. Amino acids with high identity to template were colored red. Conserved region of NBDs including Walker A/B and signature was labeled. Bottom: Sequence alignment of human MRP7 and bovine MRP1. The alignment map was generated using ESPript server.^87^

The final model is shown in Figure 1A. In this study, TMD0 was not included in our homology model due to the fact that the TMD0s in the templates were not completely resolved. Furthermore, previous studies have shown that removal of TMD0 did not affect the function of MRP1 protein.^28^ According to the sequence alignment, MRP1 and MRP7 share similar structural domains including TMDs, Lasso (L_0_ linker), and NBDs (Figure 1B–1F). The alignment contains conserved region of NBDs shared by multiple ABCC members including the Walker A, Signature, and Walker B^6^ (Figures 1D and 1F). The consensus ATPase sites are used for establishing the Mg‐ATP system.^25^

Initial models were subjected to a loop refinement procedure provided by Schrodinger Prime module. Refined MRP7 models were subjected to a series of structural assessment to determine the best model for further studies. Additionally, cryo‐EM structures of MRP1 were also evaluated with same functions. Top results are shown in Table S2. From the results, we found the homology model of MRP7 maintained high percentage of residues in Ramachandran‐favored regions (91.5% for pre‐molecular dynamics (MD) and 91.2% for post‐MD). Also, the structures got the most favored main chain layouts indicated by the three PROCHECK scores (M/c bond lengths, bond angles, and planar groups). For both pre‐MD and post‐MD, most of the residue main chain bond lengths (99.5% for pre‐MD and 99.1% for post‐MD), main chain bond angles (99.4% for pre‐MD and 98.9% for post‐MD), and planar groups (96.4% for pre‐MD and 96.0% for post‐MD) fall in reasonable range. Both crystal structures also got acceptable zDOPE, ERRAT, MolProbity, and QMEAN *Z*‐scores. The best models were selected and used for MD production runs.

Domains of MRP7 were determined according to the secondary structure of homology models as well as previous reports.^6^ Although MRP7 belongs to the “long” class of ABCC family, our model does not contain TMD0 domain due to the incompleteness of TMD0 structure in both templates.^12^ Similar to MRP1, TMD1 and TMD2 are the major TMDs of MRP7 that form the binding pocket for substrates as well as are responsible for the transportation mediated by conformational change. The two cytosolic NBDs are responsible for ATP binding and triggering the conformational change of the protein.

When considering the final refined structure after 100 ns MD runs, the Ramachandran plots of MRP7 are shown in Figure S1. The overall structure of MRP7 is maintained with 91.2% (inward‐facing) residues in most favored region (Figure S2). This result indicated that the final model retained similar quality compared with the initial model after 100 ns MD run. The following results and discussion will be based on the MD‐refined model unless otherwise stated.

**FIGURE 2:**
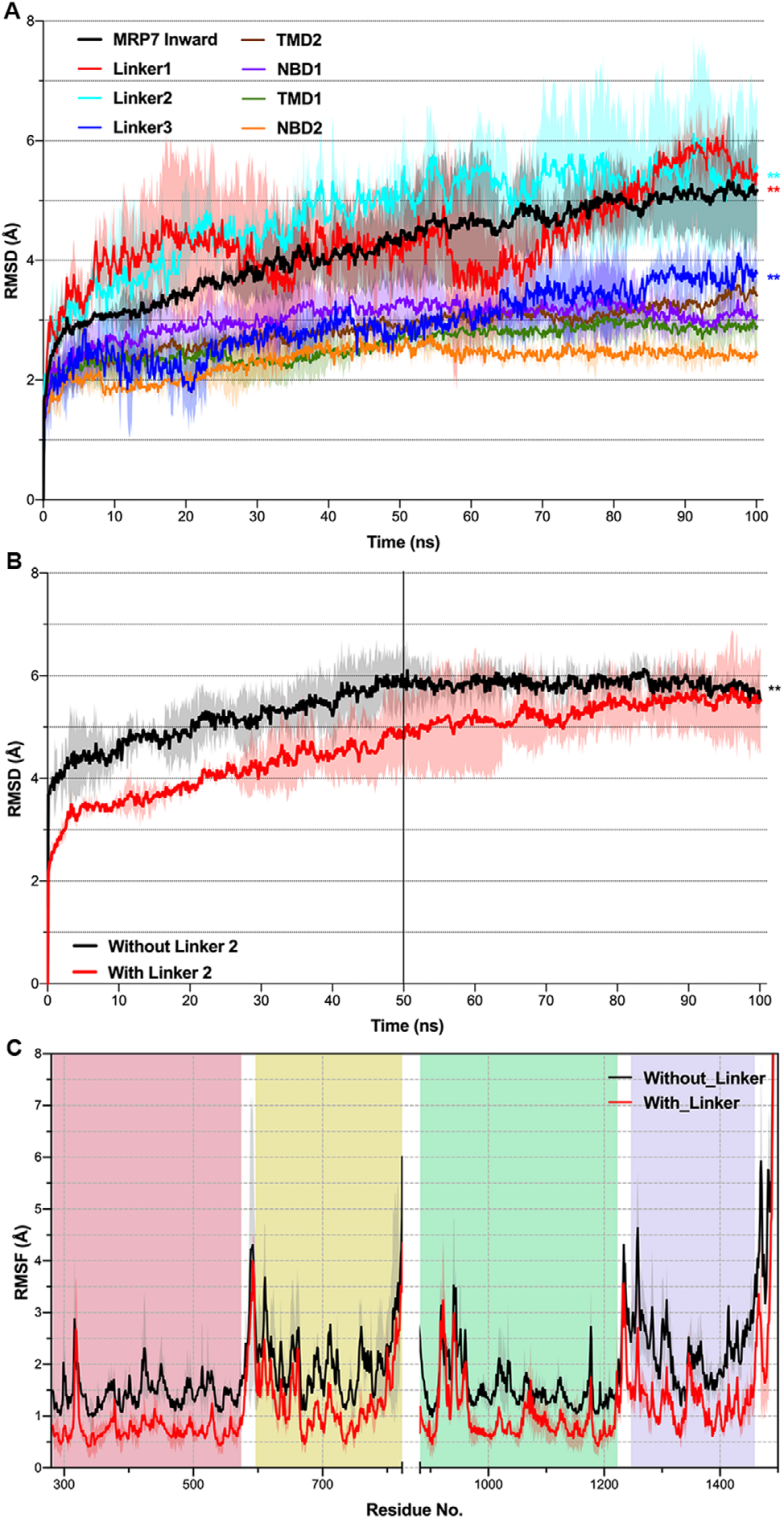
Structure deviation of MRP7 substructures in the 100 ns run. (A) The RMSDs of MRP7 substructures were plotted against time (ns). (B) RMSD of MRP7 with linker (black) or without linker (red) in 100 ns MD run. (C) RMSF of residues with linker (black) or without linker (red) in 100 ns MD run. Residue numbers were shown in *x*‐axis and segmented by domains: red, TMD1; yellow, NBD1; green, TMD2; purple, NBD2. Shadow regions indicate standard deviation of three independent MD runs. **P < 0.01 via one‐way ANOVA test compared with nonlinker structures

The structure deviation of each MRP7 domain was analyzed separately from the MD run using the root mean square deviation (RMSD). The results are displayed in Figure 2A. Overall, RMSD value of the whole MRP7 model increased in the first 50 ns, reaching a plateau of ∼5 Å until the end of the simulation. For RMSDs of separate domains, we found that the linker regions (linker 1 between TMD1 and NBD1 and linker 2 between NBD1 and TMD2) are the major contributors to the total RMSD with final RMSD at around 6 Å and relatively higher variation compared to other substructures. Linker 3 (between TMD2 and NBD2) also showed higher equilibrated RMSD compared to TMDs and NBDs, but significantly less than linker 1 and 2. TMDs and NBDs remained stable eventually at 2.5–3.0 Å.

The cryo‐EM structure of MRP1 lacks the linker 2 structure between NBD1 and TMD2 probably due to its unstable conformation as well as flexibility. Here, we analyzed the role of the de novo linker 2 structure in MRP7. Results showed that linker 2 in MRP7 contributes positively to the overall stability of the protein (Figure 2B) because the equilibrated RMSD of MRP7 without linker 2 is significantly higher than the one with linker, although its structure showed intense fluctuation and flexibility according to our RMSD data (Figure 2A). Furthermore, in the full system containing POPC membrane, missing linker resulted in increased fluctuation of residues (Figure 2C).

### 2.2 Protein global motions

**FIGURE 3:**
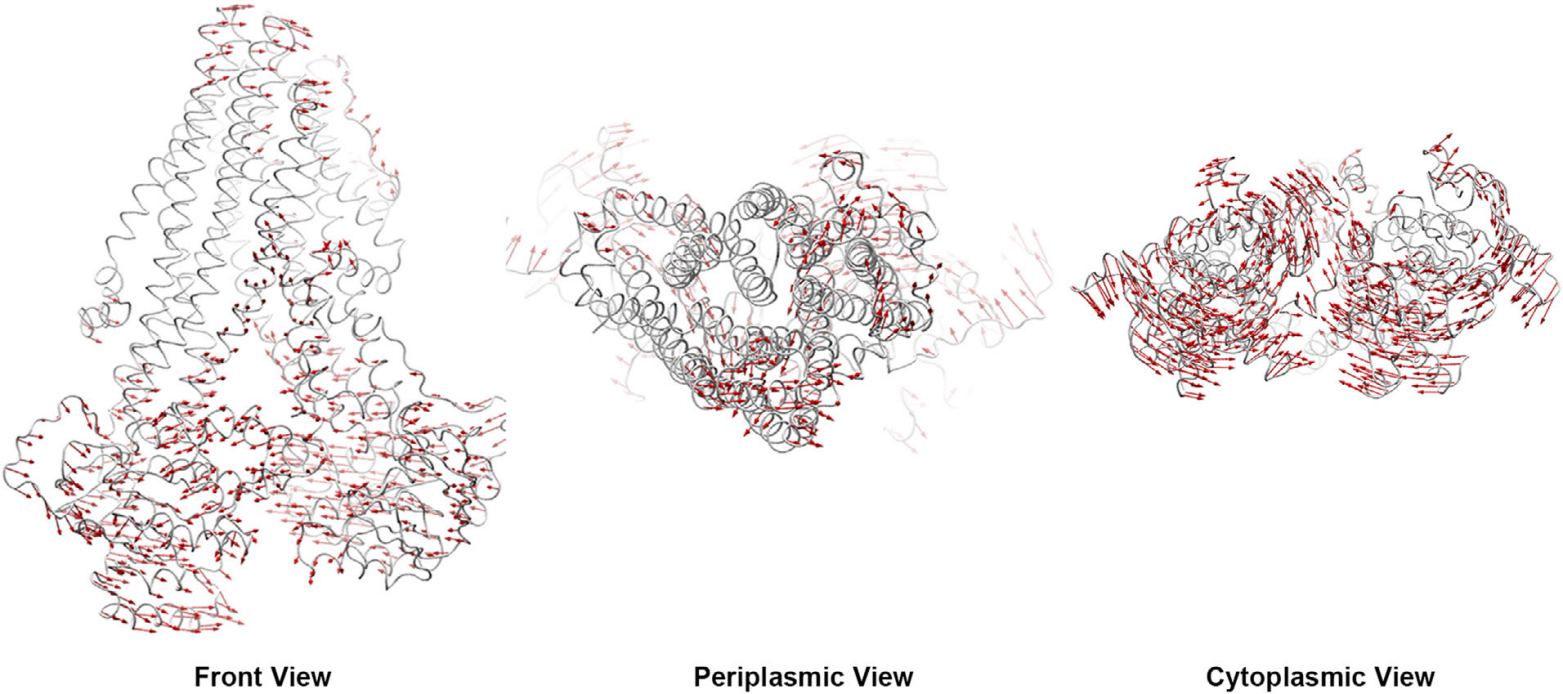
Global motion pattern of inward‐facing MRP7. Red arrows indicate potential domain motion direction and distance by PCA analysis. Motions below 1.5 Å were hidden

The protein global motion was analyzed by PCA function provided in ProDy.^29^ The major motion of protein backbone along specific directions was represented by eigenvectors derived from the covariance matrix calculated from consecutive MD trajectories.^30^ In inward‐facing MRP7, the NBDs showed higher mobility and a pendular motion parallel to the cytoplasm plane and approach each other. Also, the linker 2 structure showed opposite motion direction in upper and lower segment. Specifically, the upper segment that connects to TMD2 moved to the same direction as NBD2, whereas the lower segment moved to the same direction as NBD1. Thus, we infer that the linker absorbs forces generated in intensive movements and caused lagged equilibration and less residue fluctuation compared to when no linker 2 was present (Figure 3). The TMDs did not show as significant motions as NBDs, but slight oscillatory movements along with connected NBDs were observed. Although such movements of TMDs did not disturb the arrangement of transmembrane helices, such trend revealed the mechanism of initial motions before ATP‐binding‐triggered ligand affinity change. TMDs were connected to NBD1 and NBD2 by linker 1 and linker 3, respectively, which seemed to play a similar role in signal propagation between NBDs and TMDs as the SP domains in ABCG2.^11^

### 2.3 Binding pockets identification and validation

**FIGURE 4:**
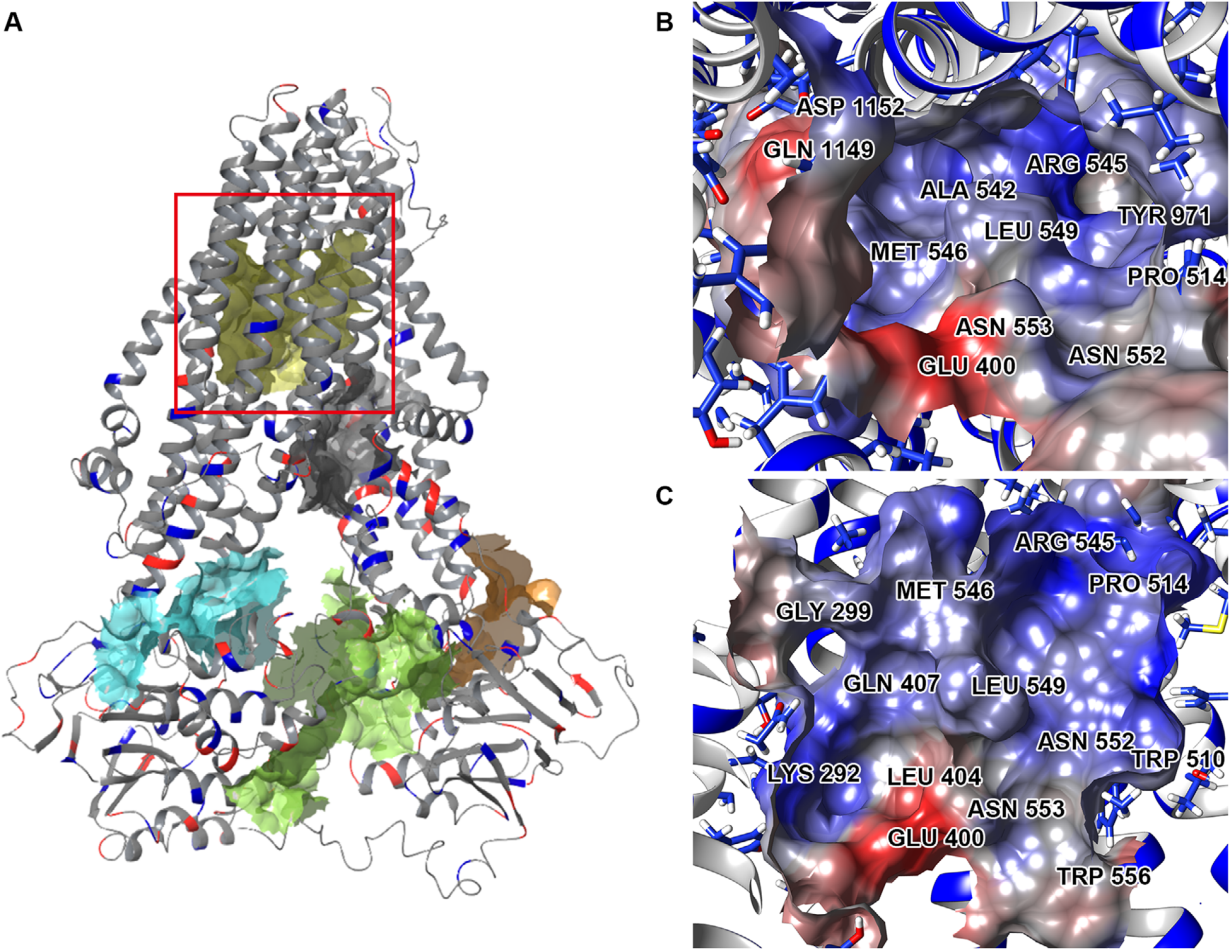
Predicted binding pockets of inward‐facing MRP7. (A) All predicted binding pockets were displayed with colored solid surface. The pocket marked by the red square was the selected binding pockets for docking analysis. (B and C) Cytoplasmic (B) and front (C) view of the marked binding pockets. Molecular surface was colored by residue electrostatic potential (red: negative; blue: positive)

To validate the function of our MRP7 model, potential binding pockets were identified (Figure 4) for docking analysis (inward‐facing). Five potential binding pockets were identified. According to Figure 4A, the yellow pocket embedded in the TMDs was the one we are interested in, because its position is consistent with the general binding pockets of ABC transporters such as ABCB1 and ABCG2, which also have functional binding pockets buried in TMDs. Other binding pockets locate either in TMDs or around NBDs. Figures 4B and 4C showed the electrostatic potential of the binding pocket as well as the shape. The volume of this pocket is around 1170 Å^3^, with the Leu549 standing out in the center and separating the pocket into two “chambers.”

**FIGURE 5:**
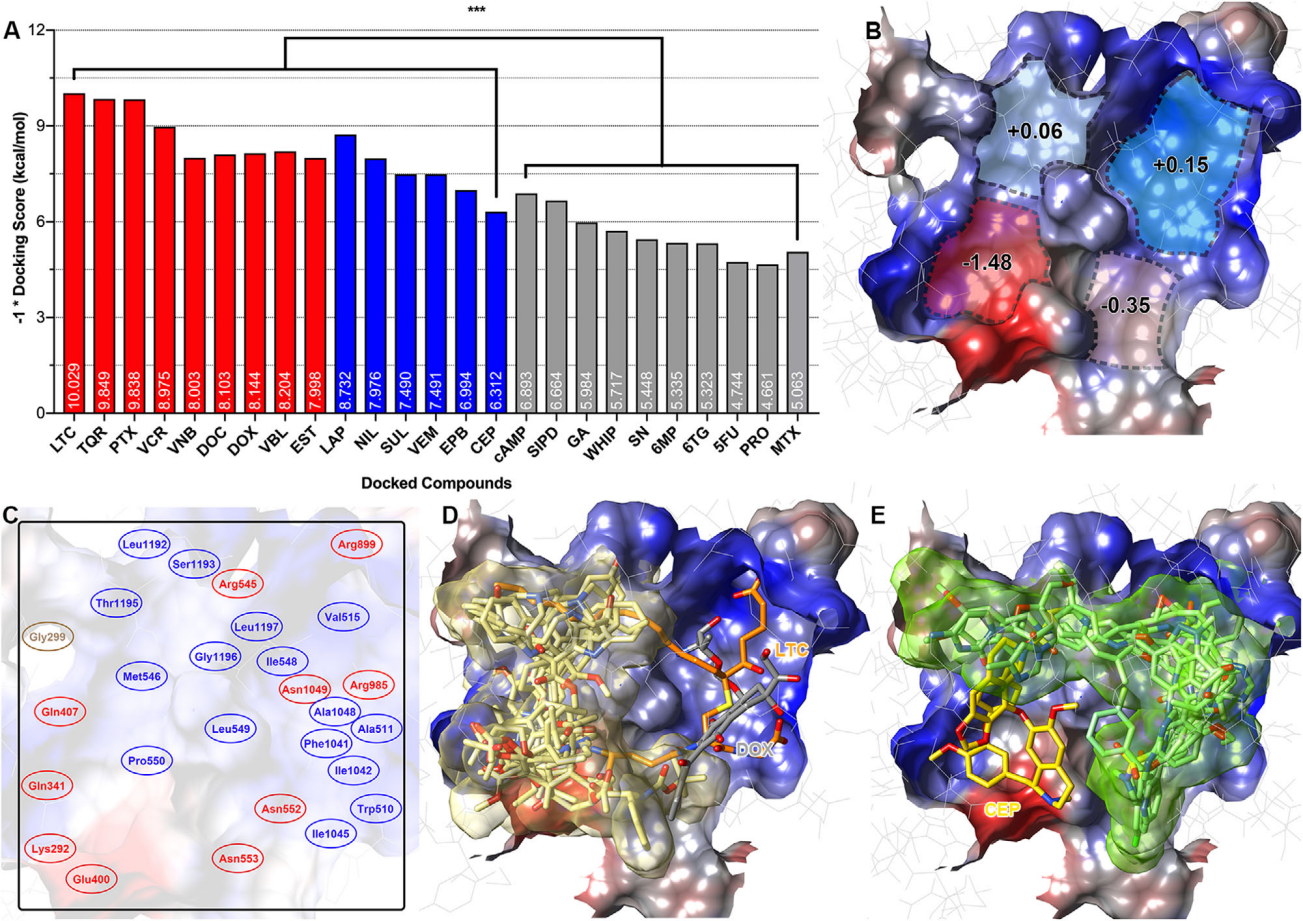
Functional validation of the MRP7 homology model by docking with experimentally validated drugs. (A) Docking scores of substrates/inhibitors/nonmodulators. Red columns indicate known MRP7 substrates; blue columns indicate known MRP7 inhibitors; gray columns indicate drugs that are not interacting with MRP7. ***P < 0.005 via one‐way ANOVA by comparing substrates/inhibitors and negative drugs. Docking scores × (–1) were labeled within columns. (B) Properties of predicted MRP7‐binding pocket. Blue indicates hydrophobic and red indicates polar. Numbers indicate mean hydrophobicity of predicted binding clefts. (C) Key residues of predicted binding pocket were labeled. Red indicates polar amino acid and blue indicates hydrophobic amino acids. Glycine is colored yellow. (D) Docked poses of MRP7 substrates in the binding pocket. LTC_4_ and doxorubicin bind to different positions as other substrates. LTC_4_ was colored orange; doxorubicin was colored gray. (E) Docked poses of MRP7 inhibitors (displayed as green sticks) in the binding pocket. Inhibitor cepharanthine was colored yellow. MRP7 protein surfaces were colored by electrostatic potential (red: negative; blue: positive) Abbreviations: LTC, LTC_4_; TQR, tariquidar; PTX, paclitaxel; VCR, vincristine; VNB, vinorelbine; DOC, docetaxel; DOX, doxorubicin; VBL, vinblastine; EST, E_2_17βG; LAP, lapatinib; NIL, nilotinib; SUL, sulfinpyrazone; VEM, vemurafenib; EPB, epothilone B; CEP, cepharanthine; SIPD, siphonellinol D; GA, glucuronic acid; WHIP, WHIP‐154; SN, SN‐38; PRO, probenecid; MTX, methotrexate.

Investigations of the transport properties of MRP7 revealed its ability to efflux conjugates such as 17β‐estradiol‐(β‐D‐glucuronide) (E_2_17βG) and leukotriene C_4_ (LTC_4_).^7^ Also, MRP7 is responsible for mediating the efflux of structurally distinct chemotherapeutic agents including doxorubicin, vincristine, docetaxel, paclitaxel, vinblastine,^31^ and vinorelbine.^32^ Moreover, our lab has previously discovered several small‐molecule drugs being MRP7 modulators including nilotinib,^19^ lapatinib,^20^ cepharanthine,^33^ sildenafil,^34^ tariquidar,^35^ epothilone B, and sulfinpyrazone,^32^ which could sensitize MRP7‐overexpressing resistant cells to substrate anticancer drugs. We also included several drugs that do not show significant interaction with MRP7 such as cAMP,^7^ siphonellinol D,^36^ glucuronic acid,^7^ WHIP‐154, SN‐38, 6‐MP, 6‐TG, 5‐FU,^31^ probenecid,^7^ and methotrexate.^6^ Thus, it is particularly interesting to look into the interactions between MRP7 and those drugs. Figure 5A showed the docking results of known MRP7 substrates, inhibitors, and those that do not interact with MRP7. The results indicated that our model could reasonably distinguish MRP7 modulators (substrates/inhibitors) and nonmodulators because the modulators (red and blue) have significantly stronger predicted affinity than nonmodulators (gray).

As mentioned above, it would be important to understand the interaction between MRP7 and its modulators. Thus, we first analyzed the docking site by clustering all docked ligands. Four separate binding clefts were predicted via EPOS^BP^ software using the docked ligands and predicted binding sites. Mean hydrophobicity was calculated based on the residue properties (Figure 5B). The best docking poses of substrates and inhibitors formed two clusters that were roughly separated by the Leu549. As shown in Figure 5, MRP7 substrates (Figure 5D) tend to bind at the hydrophilic side (yellow cluster in Figure 5D) surrounded by Gly299, Gln341, Glu400, Gln407, Leu494, Arg545, Pro550, Asn552, Asn553, Arg985, Asp1152, Thr1195, Gln1156, and Gly1196. The exceptions were LTC_4_ and doxorubicin, which have higher binding affinity in different sites. For LTC_4_, the binding pattern is consistent with its bipartite characteristic. The polar GSH moiety was stabilized by hydrogen bonds formed with Arg545, Asn552, Asn553, and Arg985, whereas the hydrophobic tail was stabilized by nonpolar interactions with Leu298, Pro303, Leu1192, and Gly1196 (Figure S7). The binding pattern of LTC_4_ with our homology model is similar to that in bovine MRP1, where LTC_4_ was also stabilized in a bipartite pocket (PDB ID: 5UJA). After summarizing the docked ligands, we proposed the key amino acids of the binding pocket displayed in Figure 5C. These key residues played crucial rules in stabilizing multiple ligands. For example, Lys292 was responsible for forming hydrogen bonds with docetaxel, doxorubicin, vinorelbine, vincristine, E_2_17βG, and cepharanthine; Leu549 was involved in stabilizing all docked ligands via hydrophobic interactions. Additionally, we found that MRP7 inhibitors tend to occupy the hydrophobic cleft (Figure 5E). Among the modulators we analyzed, cepharanthine, an herbal extract from *Stephania cepharantha*, unlike other modulators, binds at the polar site. This could be explained by the similarity in the chemical structures of cepharanthine as *Vinca* alkaloid, such as vincristine, which also binds to the polar site. Details of the docked complexes are provided in Figures S3–S15.

### 2.4 Comparative analysis with bovine MRP1

**FIGURE 6:**
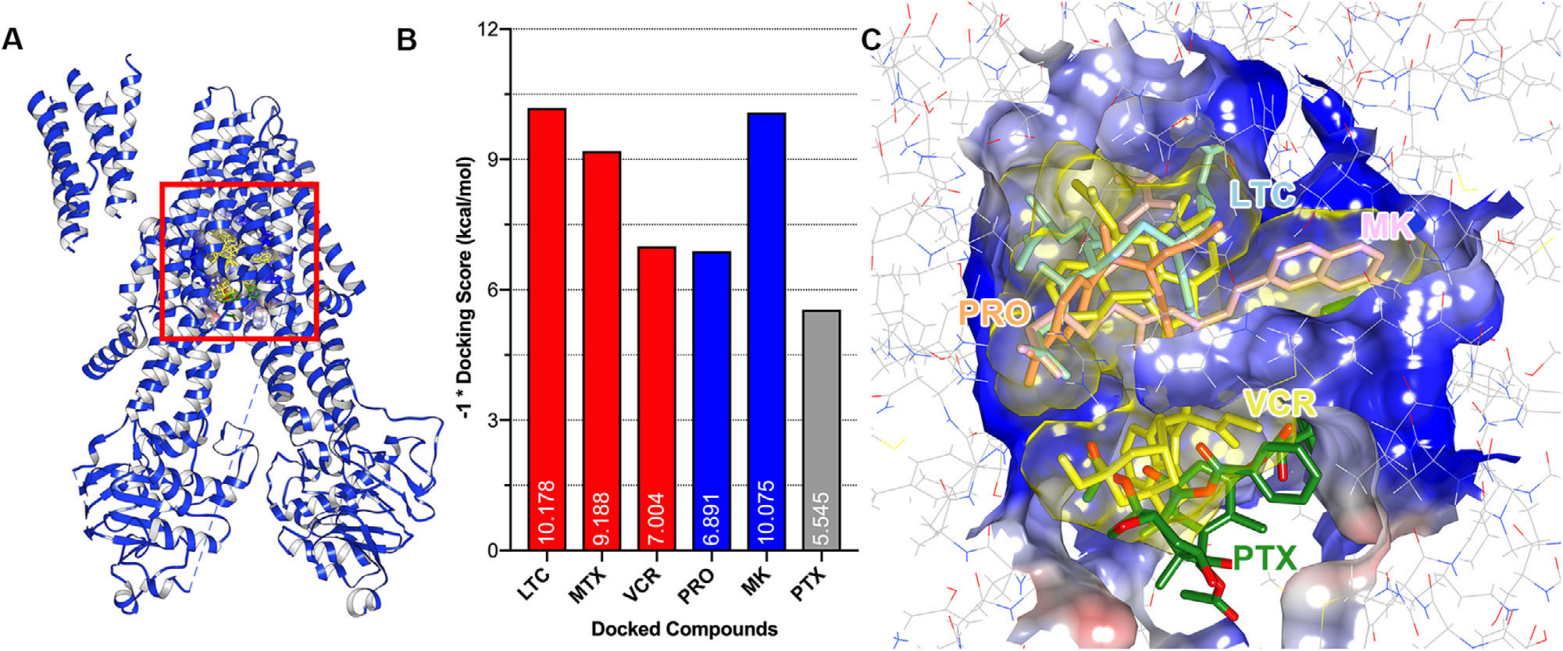
MRP1 substrate anticancer drugs and modulators docked into MRP1‐binding pocket. (A) Overview of the bovine MRP1 substrate‐binding pocket. (B) Docking scores of known MRP1 substrates, inhibitors, and non‐modulator drugs. Docking scores × (–1) were labeled within columns. Column colors indicate different types (red: substrates; blue: inhibitors; gray: non‐modulator drugs). (C) Docked poses of MRP1 substrates LTC_4_ (blue) and vincristine (yellow); modulators probenecid (orange) and MK571 (pink). Nonsubstrate paclitaxel was displayed as green sticks. Protein surface was colored by electrostatic potential (red: negative; blue: positive). Abbreviations: LTC, LTC_4_; PTX, paclitaxel; VCR, vincristine; MTX, methotrexate; PRO, probenecid; MK, MK571.

Next, we performed a series of docking simulations to compare the ligand‐binding patterns of MRP7 and MRP1. Multiple anticancer drugs have been identified as MRP1 substrates (such as vincristine and methotrexate) or inhibitors (such as probenecid and MK571). Previous studies have demonstrated that methotrexate is a substrate of MRP1 but not MRP7.^6^, ^53^ Similarly, paclitaxel is a good substrate of MRP7 but it cannot be transported by MRP1.^6^, ^54^ In this section, we first performed docking analysis using LTC_4_‐bound bovine MRP1 structure. Results are shown in Figure 6. In Figure 6A, we found that the drug‐binding pocket of MRP1 was also buried in TMDs. Details of the binding pocket are given in Figure 6C. Different from MRP7, MRP1 has a relatively smaller binding pockets with volume of around 743 Å^3^. Figure 6B showed the results of docking simulations using known MRP1 substrates/inhibitors and nonmodulators. The results are consistent with previous in vitro studies, with substrates and inhibitors showing higher scores than nonsubstrate paclitaxel. Detailed docked complex of ligand–MRP1 can be found in Figure S16–S19.

**FIGURE 7:**
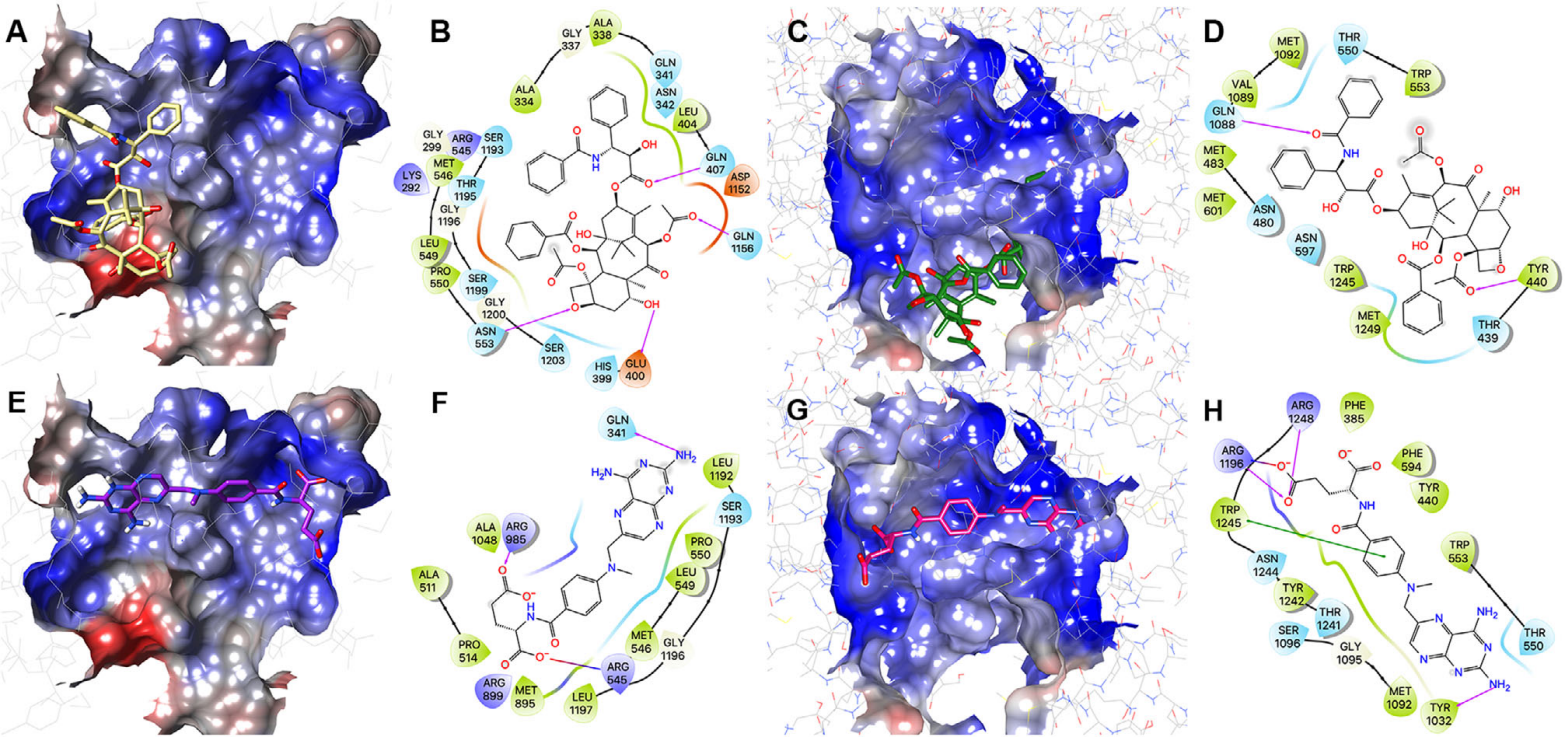
Paclitaxel/methotrexate docked with MRP7‐ and MRP1‐binding pockets. (A) Paclitaxel docked with the MRP7‐binding pocket. Protein surface was colored by electrostatic potential. Paclitaxel was displayed as yellow sticks. (B) Two‐dimensional diagram of paclitaxel–MRP7 interactions. Amino acids within 3 Å to paclitaxel were displayed as colored bubbles (cyan: polar; green: hydrophobic; red: negatively charged; blue: positively charged). Purple lines with arrow indicate hydrogen bonds. Gray circles indicate solvent exposure. (C) Paclitaxel docked with the MRP1‐binding pocket. (D) Two‐dimensional diagram of paclitaxel–MRP1 interactions. (E) Methotrexate docked with the MRP7‐binding pocket. Protein surface was colored by electrostatic potential. Methotrexate was displayed as purple sticks. (F) Two‐dimensional diagram of methotrexate–MRP7 interactions. (G) Methotrexate docked with the MRP1‐binding pocket. Methotrexate was displayed as magenta sticks. (H) Two‐dimensional diagram of methotrexate–MRP1 interactions. Purple solid lines with arrow indicate hydrogen bonds. Purple solid line without arrow indicates salt bridge. Green solid line without arrow indicates pi–pi interaction

From Figure 6C, we found that the best docked pose of paclitaxel was actually on the edge of the pocket and close to the open end form by TMDs of MRP1. Detailed docking complex is shown in Figures 7C and 7D. For MRP7, the paclitaxel was stabilized by both polar and hydrophobic interactions. From Figure 7B, we could see that four hydrogen bonds were formed between paclitaxel and Glu400, Gln407, Asn553, and Gln1156. The benzene rings were stabilized via hydrophobic interactions with Ala334/338, Gly333/337, Gly1196, and Leu1197. For MRP1, in Figure 7D, we could see that paclitaxel was stabilized via two hydrogen bonds with Gln1088 and Tyr440. No strong hydrophobic interaction in MRP1 was observed to stabilize the molecule. Thus, it is reasonable that paclitaxel has weak or no interaction with MRP1 in actual biological systems.

The predicted binding poses of methotrexate in MRP1/MRP7‐binding pockets are shown in Figure 7. In Figures 7E and 7F, the best docking poses of methotrexate located majorly in the two hydrophobic clefts of MRP7, thus less likely to form hydrogen bonds with polar residues to stabilize the carboxyl groups as in MRP1 (Figures 7G and 7H). According to the cryo‐EM structure of bovine MRP1, its binding pocket is actually composed of a “P‐pocket” (polar, formed majorly by Lys332, His335, Leu381, Arg1196, Arg1248, and Asn1244 and aromatic residues Phe594, Tyr440, and Phe385) and an “H‐pocket” (hydrophobic, formed majorly by Trp1245, Trp553, Met1092, and Thr550 and aromatic residue Tyr1242) based on the binding pattern of LTC_4_.^25^ Our docking results of methotrexate stay consistent with the proposed MRP1‐binding pocket. Similar to LTC_4_, methotrexate was stabilized by both polar and hydrophobic interactions. The carboxyl groups formed three hydrogen bonds with a salt bridge with Arg1196. Moreover, Trp1245 formed a pi–pi stacking interaction with the ring structure of methotrexate. Although several studies have confirmed methotrexate being a good substrate of MRP1 but not MRP7, we validated such conclusion in this study to further demonstrate the reliability of our homology model. Cytotoxicity of methotrexate was determined in transfected HEK293/pcDNA3.1, HEK293/MRP1, and HEK293/MRP7 cells. Results are shown in Table 1. The results showed HEK293/MRP1 with significantly higher IC_50_ of methotrexate than HEK293/pcDNA3.1 cell line; however, the IC_50_ of methotrexate did not show significant difference. Additionally, paclitaxel showed significant higher IC_50_ in HEK293/MRP7 cells but not in HEK293/MRP1 cells. Vincristine showed higher IC_50_ in both transfected cells. In conclusion, our docking analysis results were consistent with in vitro studies regarding the transport of methotrexate by MRP1 but not by MRP7.

**TABLE 1:** Cytotoxicity of methotrexate, vincristine, or paclitaxel in MRP1‐ or MRP7‐transfected HEK cells

	Methotrexate		Vincristine		Paclitaxel	
Cell line	IC_50_ ^a^ (μM)	FR^b^	IC_50_ ^a^ (nM)	FR^b^	IC_50_ ^a^ (nM)	FR^b^
HEK293/pcDNA3.1	2.23 ± 0.43	1.0	7.24 ± 0.91	1.0	6.28 ± 0.88	1.0
HEK293/MRP1	12.22 ± 3.49^*^	5.5	87.33 ± 10.13^*^	12.1	6.43 ± 0.23	1.0
HEK293/MRP7	3.97 ± 0.33	1.8	70.45 ± 8.33^*^	9.7	72.36 ± 9.14^*^	11.5

*Note*: IC_50_ is presented as IC_50_ value ± SD; FR, fold resistance.

^a^ Calculated from at least three independent experiments with triplicates.

^b^ Resistance folds were determined by division of IC_50_ in HEK293/pcDNA3.1 cells versus MRP1‐ or MRP7‐transfected cells.

* *P* < 0.05. HEK293/pcDNA3.1 versus MRP1‐ or MRP7‐transfected cells.

## 3 DISCUSSION

Because functional modulation of the ABC transporters is considered a major solution in overcoming MDR in cancers,^39^ understanding the structure–function relationship is thus important in the development of novel ABC transporters modulators. Moreover, information in drug binding and recognition based on structural analysis is necessary in the discovery of potential ABC transporters substrates^40^, ^41^ that will provide valuable references for clinicians. In the past decades, tremendous efforts have been made to circumvent the acquired chemoresistance including designing/synthesizing novel selective ABC transporter modulators,^43^, ^44^, ^45^, ^46^, ^47^ repurposing small molecule drugs,^57^, ^64^, ^65^, ^66^, ^67^, ^68^, ^69^, ^70^, ^71^, ^72^, ^73^ or peptides^58^, ^59^, ^60^ as well as discovering potential ABC transporters substrates that have been used in clinic in order to prevent the decrease in therapeutic effects by adjusting treatment strategies.^42^, ^61^ MRP7 is an important member in ABCC subfamily with wide expression in tissues and is responsible for mediating MDR against multiple chemotherapeutic drugs.^62^

Considering the lack of MRP7 crystal structure, we constructed two conformations of human MRP7 models using homology modeling tools and equilibrated by a 100 ns MD run. Subsequently, the structural dynamics was analyzed based on the MD simulation, where we found that linker structures, especially linker 1 and linker 2, were the major contributors to the overall structural deviation. In the most recent crystal structures of ABCC transporters, these linker domains were unable to fully determined by crystallography,^25^, ^26^, ^79^ which is collaborated with the flexibility of these particular regions in our MD run.

The structure of the longest linker 2 that connects TMD2 and NBD1 often lacks accurate three‐dimensional structure in ABC transporter crystal structures. In this study, we constructed de novo linker structure and evaluated the role it plays in the structural dynamics in MRP7 via RMSD and RMSF analysis. Our results indicated that the linker structure stabilized the protein structure by connecting the two functional complexes (TMD1–NBD1 and TMD2–NBD2) as a “spring” in order to maintain the protein structure and transmit domain motions. Similarly, in other ABC transporter structures such as ABCB1, the linker structure also lacks accurate crystal structure.^64^ The linker structure of ABCB1 was found by a homology modeling and MD simulation study to stabilize the transporter in membrane system.^65^ The MD results in Figure 2A that TMD2 and NBD1 having higher equilibrated RMSD could be explained by direct connection with linker 2. The fluctuation of linker 2 will be transmitted to domains that are directly linked more easily. And with the increase in distance to linker 2, its influence in structural stability will become attenuated.

Being an ATP‐dependent efflux pump, analysis of the potential motion pattern of MRP7 will boost the understanding of the transportation mechanism. The PCA analysis revealed the potential motions of MRP7 at substrate‐binding or ATP‐binding states. Several studies on other ABC transporters have described essential mechanism of action related to the NBD dimerization after substrates and ATP binding.^26^, ^80^, ^82^ For MRP1, the cryo‐EM structures of inward‐ and outward‐facing indicate that the ATP binding induces NBD closure that consequently triggers helices rotation and side chain movement as well as decreased substrate affinity.^26^

Subsequently, we performed binding site search as well as docking analysis to characterize the binding pockets of MRP7, where the major drug‐binding pocket buried in TMDs was focused. The binding pockets of MRP1 and MRP7 were also compared and analyzed. Unlike ABCB1, where the binding sites are characterized by a large number of aromatic (M site) and polar (R/H site) residues,^67^ and ABCG2, where the binding sites are majorly composed of hydrophobic residues,^11^ both MRP7 and MRP1 showed bipartite‐binding pockets with the existence of both polar and nonpolar regions. Although sharing the similar components, MRP7 and MRP1 still have different substrate/inhibitor spectrum; for example, multiple studies have shown that MRP1 is not able to mediate the transportation of paclitaxel,^68^, ^69^ which is a substrate of MRP7,^70^ methotrexate, which is a substrate of MRP1 but not MRP7. Such difference could be explained by the characteristics of the pockets such as volume and hydrophobicity. We found that paclitaxel had lower binding affinity to MRP1 possibly due to the size of the pocket and distribution of polar residues. As a result, paclitaxel could only find the best docking site on the edge of the pocket, which has polar residues and larger volume. It is worth noting that although the different physiochemical properties of amino acids that form the binding pocket of MRP7 and MRP1 are one of the key factors that determined the transport pattern of paclitaxel, paclitaxel sensitivity is also determined by other intracellular macro‐factors as well as metabolism pathways.

Investigations of the transport properties of MRP7 revealed its ability to efflux conjugates such as E_2_17βG and LTC_4_,^7^ indicating the existence of bipartite substrate‐binding pocket for anionic and hydrophobic moieties. In this study, we identified four potential binding clefts with different hydrophobicity in MRP7‐binding pockets, which were separated by a leucine in the center. The key amino acids that are involved in ligand binding are also summarized in Figure 7B. The docking analysis of several previously validated MRP7 substrates provided more details of the binding pocket. More docking analysis was performed using more MRP7 substrates and modulators, from which we further confirmed the existence of binding clefts. Additionally, we found that MRP7 modulators tend to occupy the hydrophobic cleft, whereas substrates tend to occupy the polar cleft. Among the modulators we analyzed, cepharanthine, an herbal extract from *Stephania cepharantha*, unlike other modulators, binds at the polar site. This could be explained by the similarity in the chemical structures of cepharanthine as *Vinca* alkaloid, such as vincristine, which also binds to the polar site.

In summary, we performed a series of structure–function analysis using MD simulations and docking on homology models built from MRP1 crystal structures. Our findings provide new and valuable information for better understanding the structural dynamics and transport mechanism of human MRP7, as well as the potential drug‐binding sites within the TMDs of MRP7. Our model was also validated by docking analysis using known MRP7 substrates and inhibitors, as well as nonmodulators. This MRP7 model could be a good starting point for future MRP7 studies regarding amino acid mutations in cancer patients to evaluate potential alterations of substrates/inhibitors binding pattern and pharmacokinetics. Moreover, our model would theoretically enable the development of MRP7 modulators as well as high‐throughput virtual screening.

## 4 MATERIALS AND METHODS

### 4.1 Homology modeling of human MRP7 and structure refinement

Human MRP7 sequence (validated in vitro) was obtained from the publication by Hopper et al., in which human MRP7 was first discovered and expressed.^6^ Before modeling, a BLAST (basic local alignment search tool) search was performed on PDB by using MRP7 protein sequence to find suitable templates. Bovine MRP1 proteins (5UJA) were selected as templates considering the identity and resolution.

A common homology modeling procedure includes template alignment, alignment adjustment, backbone establishment, loop/side chain prediction, and model refinement.^71^ In this study, we used the homology modeling tool Prime provided by Schrodinger Suite and visualized in Maestro 11 (Schrödinger, NY). The TMD0 region of MRP7 was eliminated before alignment. For each conformation, we created 50 initial models (totally 100) followed by loop refinement provided in Schrodinger Prime and subjected to quality assessment.

### 4.2 Protein structure assessment

Generated homology models were evaluated for structure integrity to select the one with best quality as judged by Ramachandran‐favored residues, main chain (M/c) bond lengths and bond angles,^72^ peptide bond planarity,^73^ and zDOPE (normalized Discrete Optimized Protein Energy) score.^74^ Models with zDOPE score closer to –1.0 have better quality. The Ramachandran plots, M/c bond lengths/angles, and peptide bond planarity scores were calculated using PROCHECK.^75^ Furthermore, the QMEANBrane^76^ function was applied to better assess transmembrane protein model quality. Moreover, QMEAN Z‐score,^77^ ERRAT,^78^ and MolProbity^79^ scores were also calculated. In brief, (1) QMEAN *Z*‐score evaluates how a protein model is in agreement with the one expected from experimental structures of similar size. The *Z*‐score is an integration of global (QMEAN4) and local (QMEAN6) estimates of protein quality. Scores closer to zero indicate good structural quality.^77^ (2) ERRAT scores describe the overall quality of a protein model and higher score indicates better quality. The normally accepted range for ERRAT score is >50.^78^ (3) MolProbity score is a combined protein quality score that indicates the expected resolution of a possible crystal structure of similar quality with tested protein model. Thus, lower MolProbity score means better quality.^79^

### 4.3 Membrane system and MD simulations

MD simulation was set up and performed as previously described with modifications.^80^ MD simulation system was built using the system builder tool provided in Desmond (D.E. Shaw Research, NY). The membrane systems for inward‐facing MRP7 were built separately. A POPC membrane with a predefined TIP3P solvent model was established for simulation run. Na^+^ and Cl^−^ ions were added to neutralize the overall charge of the system. The MD simulation was performed in periodic boundary conditions. After a default relaxation protocol, the simulation was performed as *NpT* runs used Nose–Hoover thermostat^81^ and Martyna–Tobias–Klein barostat^82^ methods with isotropic coupling under temperature 300 K and pressure 1 bar for 100 ns. All MD runs for substructure dynamics analysis were performed independently for three times with different random seeds. All simulations were performed in Ubuntu 18.04 system with an NVIDIA Tesla P100 GPU. In total, 1000 frames were generated and subjected to a principal component analysis (PCA) for protein motion pattern prediction using ProDy^29^ with in‐house python (3.6) scripts and visualized through the NMWiz plugin in VMD.^83^

### 4.4 Binding sites identification

Inward‐facing human MRP7 homology model was prepared for binding site search as previously described.^84^, ^85^ In brief, the protein model was preprocessed using the Protein Preparation Wizard provided in Schrodinger Suite. Preprocessing steps include adding hydrogen atoms, assigning bond orders, and removing water molecules. Binding pockets were identified using SiteMap in Schrodinger Suite and visualized in Maestro 11 (Schrödinger, NY).

### 4.5 Molecular docking

Docking simulations were performed using AutoDock Vina (1.1.2).^86^ The protein model and the ligands were modified by adding hydrogen atoms and partial charges in AutoDockTools (1.5.4). Docking grid center and size were determined according to the binding pocket surrounded by TMDs of MRP7 as well as MRP1 (MRP1‐binding pocket was determined by co‐crystalized ligand). Specifically, the docking grid was determined by the center coordinates of the predicted binding region. The size of the grid box is 30 Å × 30 Å × 30 Å. Each docking run generated 10 poses with the highest docking score. All other parameters were set as default. The ligands with highest affinity score were exported for visualization and further analysis. The two‐dimensional interaction diagram was generated by Maestro provided by Schrodinger. The three‐dimensional ligand–protein figure was generated using UCSF Chimera (v.1.14). Protein surface was colored by electrostatic potential calculated by the Coulombic Surface Coloring module.

### 4.6 Binding pocket characterization

Volume of the MRP1 and MRP7 drug‐binding pockets was calculated using SiteMap provided by Schrodinger. For hydrophobicity, we randomly sampled 50–60 amino acids with docked/crystalized ligands in the center. We used Kyte–Doolittle amino acid hydrophobicity scale (KD hydrophobicity score) to calculate the overall hydrophobicity of the pockets. Weighted hydrophobicity scores were calculated by

$$\frac{\mathrm{Number}\;\mathrm{of}\;\mathrm{selected}\;\mathrm{residue}}{\mathrm{Number}\;\mathrm{of}\;\mathrm{sampled}\;\mathrm{residues}}\times \;\mathrm{KD}\;\mathrm{hydrophobicity}\;\mathrm{score}.$$

### 4.7 Cell lines and chemicals

HEK293/pcDNA3.1, MRP1‐transfeced HEK293/MRP1, and MRP7‐transfeced HEK293/MRP7 cell lines were cultured in DMEM with 10% fetal bovine serum at 37°C with 5% CO_2_. Transfected cells were established and maintained as previously described.^31^ Vincristine, paclitaxel, and methotrexate were purchased from Sigma Chemical Co.

### 4.8 Cell viability assay

Cytotoxicity of methotrexate was determined in MRP1‐ or MRP7‐overexpressing cell lines using the MTT assay as described previously.^35^ Briefly, equal number of cells were seeded in 96‐well plates 24 h prior to drug treatment. After incubation in culturing environment for 68 h, MTT was added and incubated for extra 4 h. DMSO was added in each well to dissolve the purple formazan. Absorbance at 570 nm was measured and IC_50_ values were calculated as previously described.^35^

### 4.9 Statistical analysis

In this study, we performed statistical analysis to evaluate the difference of (1) MD simulations for substructural dynamics analysis; (2) comparing the overall RMSD of MRP7 with or without linker 2; and (3) comparing the hydrophobicity of MRP1‐ and MRP7‐binding pockets. Only trajectories after equilibration (50 ns) were considered for statistical analysis. In vitro MTT assay in this study was generated from at least three independent triplicated experiments. Results were presented as mean ± SD. All pair‐wise comparisons were performed using one‐way ANOVA followed by post hoc analysis.

## ADDITIONAL INFORMATION

The Supporting Information contains the coordinates of the final protein model docked with paclitaxel, quality assessment results, Ramachandran plots, and chemical structures of small molecule drugs used in our docking analysis. Docked complex of MRP7 modulators were also displayed in the Supporting Information.

## AUTHOR CONTRIBUTIONS

The manuscript was written and prepared entirely by JQW and ZSC. JQW performed the image processing, modeling, and molecular dynamics experiments. QC, ZNL, QXT, ZL, and NJ contributed to the experiments, experimental design, and discussions. JQW, LL, ZL, and ZSC contributed to the overall design of the project and discussions.

## CONFLICT OF INTEREST

The authors declare no conflict of interest.

## ETHICS STATEMENT

Not applicable.

## Supporting information

Supporting information

## Data Availability

The data that support the findings of this study are available from the corresponding author upon reasonable request.
