# Supplementary material for: Insights on the structure–function relationship of human multidrug resistance protein 7 (MRP7/ABCC10) from molecular dynamics simulations and docking studies
Source: MedComm (2020). 2021 Mar 25;2(2):221–35. doi: 10.1002/mco2.65 (PMC8491190; doi:10.1002/mco2.65)
Supplement: Supplementary file 1 — Supporting information [file MCO2-2-221-s001.pdf]

Insights on the Structure-Function Relationship of Human Multidrug Resistance Protein 7 (MRP7/ABCC10) from Molecular Dynamics Simulations and Docking Studies

Jing-Quan Wang [a], Qingbin Cui [a,b], Zi-Ning Lei [a], Qiu-Xu Teng [a], Ning Ji [a], Lusheng Lin [c], Zhijun Liu [*, d], Zhe-Sheng Chen [*, a]

[a] *Department of Pharmaceutical Sciences, College of Pharmacy and Health Sciences, St. John's University, Queens, NY 11439, USA*
[b] *School of Public Health, Guangzhou Medical University, Guangzhou 511436, Guangdong, China*
[c] *Cell Research Center, Shenzhen Bolun Institute of Biotechnology, Shenzhen, Guangdong, 518118, China.*
[d] *Department of Medical Microbiology, Weifang Medical University, Weifang, 261053, China*

*Address correspondence to this author at the Department of Pharmaceutical Sciences, St. John's University, Queens, NY 11439, US; E-mails: chenz@stjohns.edu (Zhe-Sheng Chen); Department of Medical Microbiology, Weifang Medical University, Weifang, 261053, China; E-mails: zhijun.liu@wfmc.edu.cn (Zhijun Liu)

SUPPLEMENTAL MATERIALS

Table. S1. Top alignments of MRP7

PDB ID	Title	Length	Identity (%)	Gaps	E value	Resolution (Å)
6BHU	Chain A, Multidrug resistance-associated protein 1 [Bos taurus]	1659	**34.80%**	9.13%	0.0	3.14
5UJA	Chain A, Multidrug resistance-associated protein 1 [Bos taurus]	1460	**34.88%**	9.13%	0.0	3.34
6C3O	Chain E, ATP-binding cassette sub-family C member 8 [Homo sapiens]	1581	28.88%	12.88%	0.0	3.90
5WUA	Chain E, SUR1 [Mesocricetus auratus]	1582	30.89%	11.89%	0.0	5.60
5YKE	Chain B, ATP-binding cassette sub-family C member 8 isoform X2 [Mesocricetus auratus]	1582	30.89%	11.89%	0.0	4.11
5TWV	Chain B, ATP-binding cassette sub-family C member 8 [Cricetus cricetus]	1590	30.74%	11.89%	0.0	6.30

Table. S2. Structure assessment of top models

	Ramachandran favored [up to 100%]	M/c bond lengths [up to 100%] [1]	M/c bond angles [up to 100%] [2]	Planar groups [up to 100%] [3]	zDOPE [down to -1]	ERRAT [up to 100]	MolProbity [down to 0]	QMEAN Z-score [up to 0]
Inward-facing models								
Pre-MD	91.5%	99.5%	99.4%	96.4%	-0.64	89.33	3.85	-1.65
Post-MD	91.2%	99.1%	98.9%	96.0%	-0.79	77.72	3.26	-1.96

[1] : percentage of main chain bonds within the allowed range
[2] : percentage of main chain angles within the allowed range
[3] : percentage of peptide bonds within the allowed planarity range

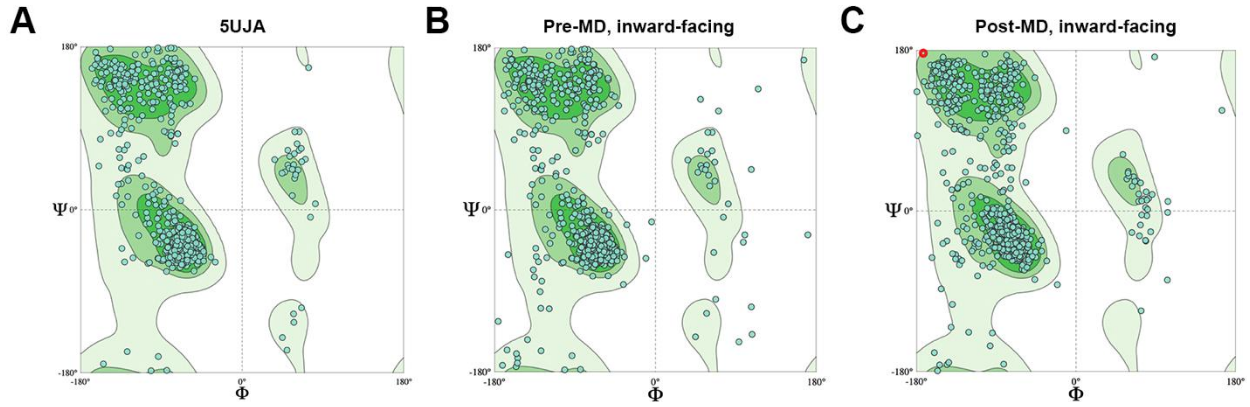

Fig. (S1). Ramachandran plots of template crystal structure 5UJA (A), initial model (B), MRP7 at the end of 100 ns MD run (C).

Table S2. Structures and docking scores of MRP7 substrate anticancer drugs and modulators.

Type	Name	Structure
MRP7 substrate anticancer drugs	Doxorubicin	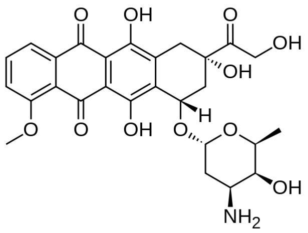
	Vincristine	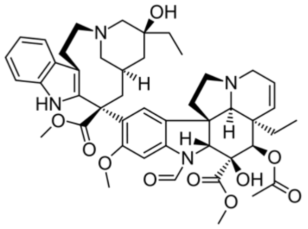
	Paclitaxel	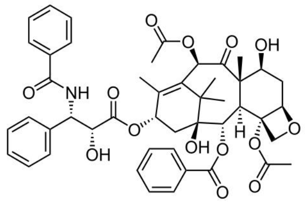
	Docetaxel	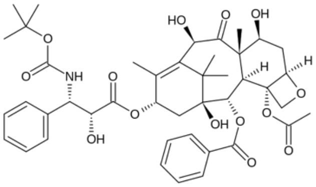

	Vinblastine	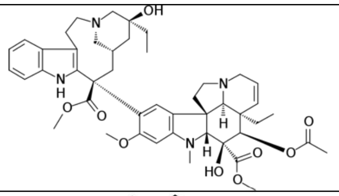
	Vinorelbine	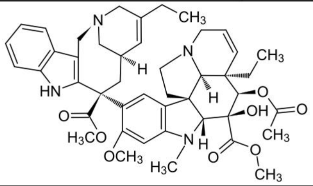
MRP7 modulators	Tariquidar	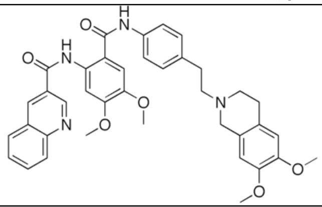
	Vemurafenib	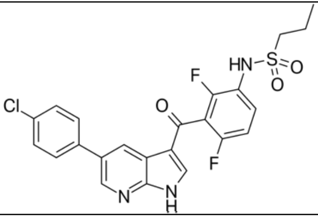
	Lapatinib	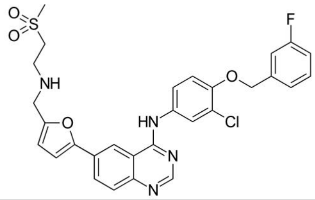
	Cepharanthine	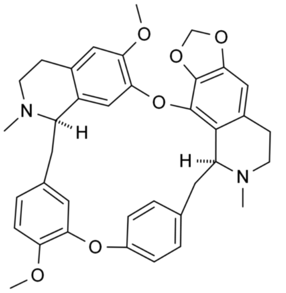
	Sildenafil	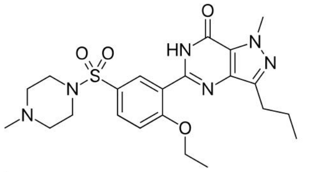
	Nilotinib	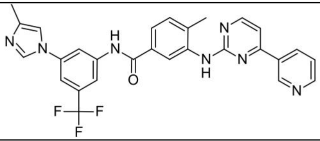

MRP7 non-interactors	Methotrexate	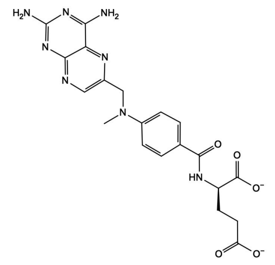
	Probenecid	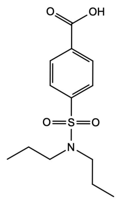
	WHIP-154	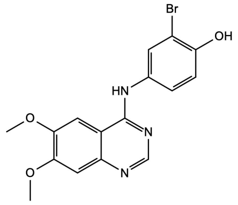
	Siphonellinol D	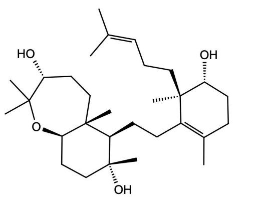
	Glucuronic acid	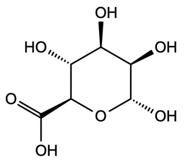
	cAMP	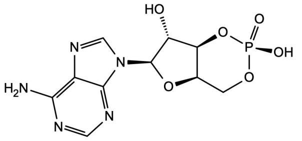
	SN-38	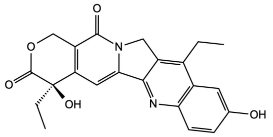
	5-FU	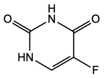
	6-MP	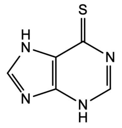

	6-TG	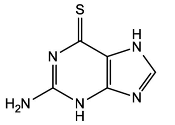

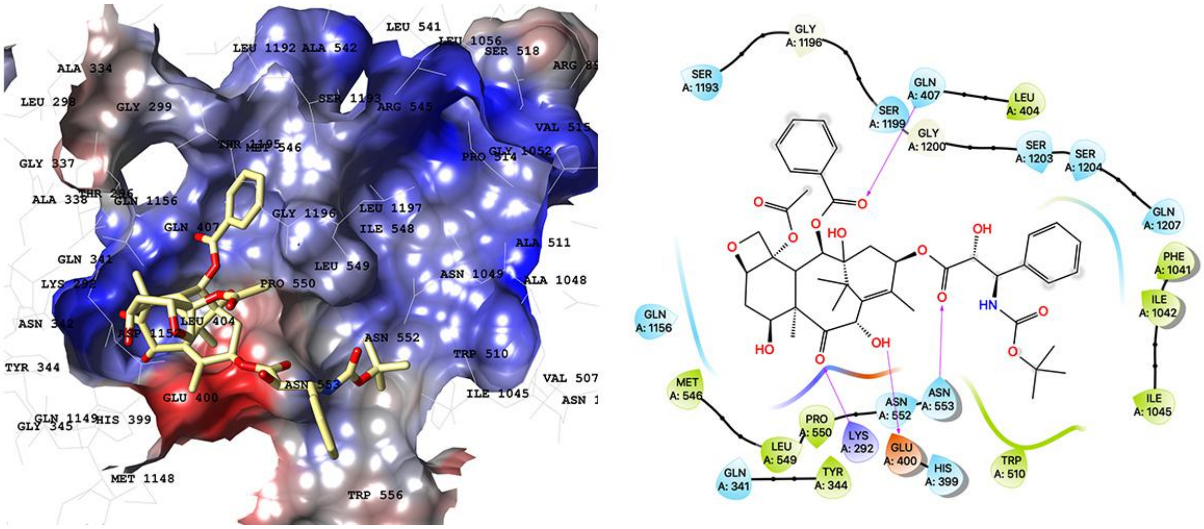

Fig. (S2). Docetaxel-MRP7 complex.

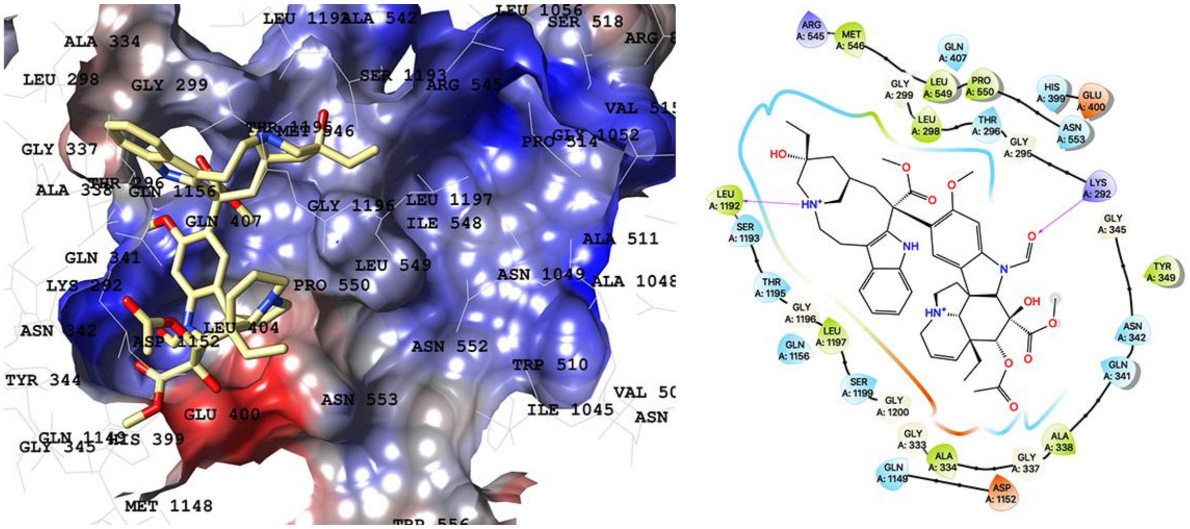

Fig. (S3). Vincristine-MRP7 complex.

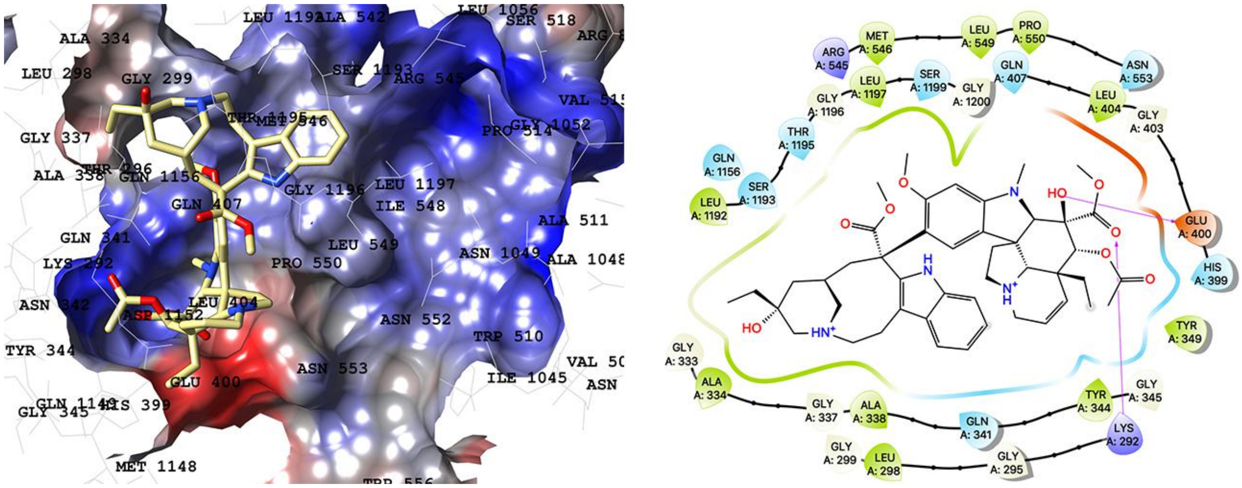

Fig. (S4). Vinblastine-MRP7 complex.

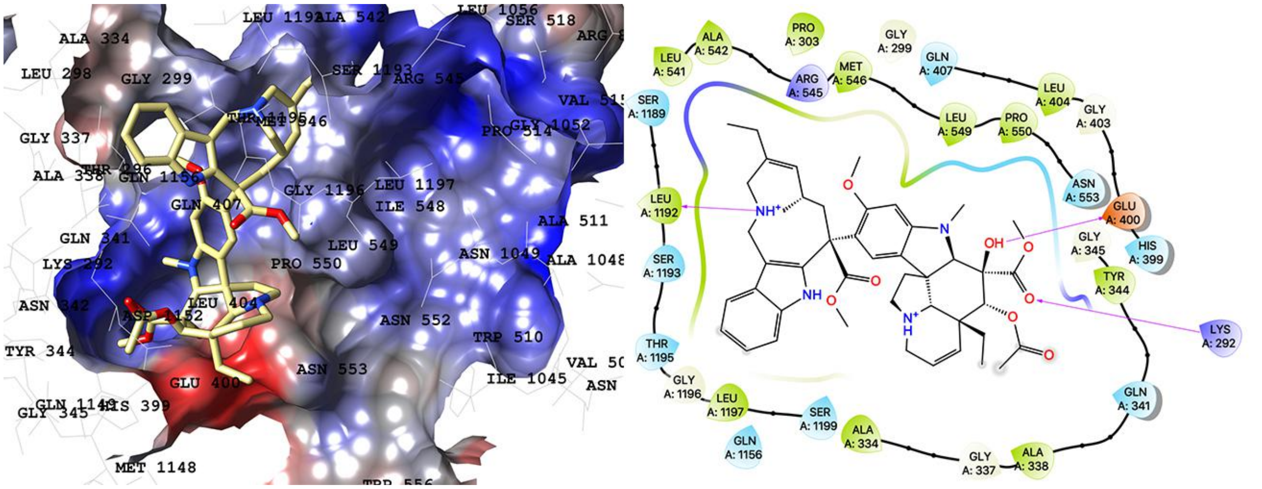

Fig. (S5). Vinorelbine-MRP7 complex.

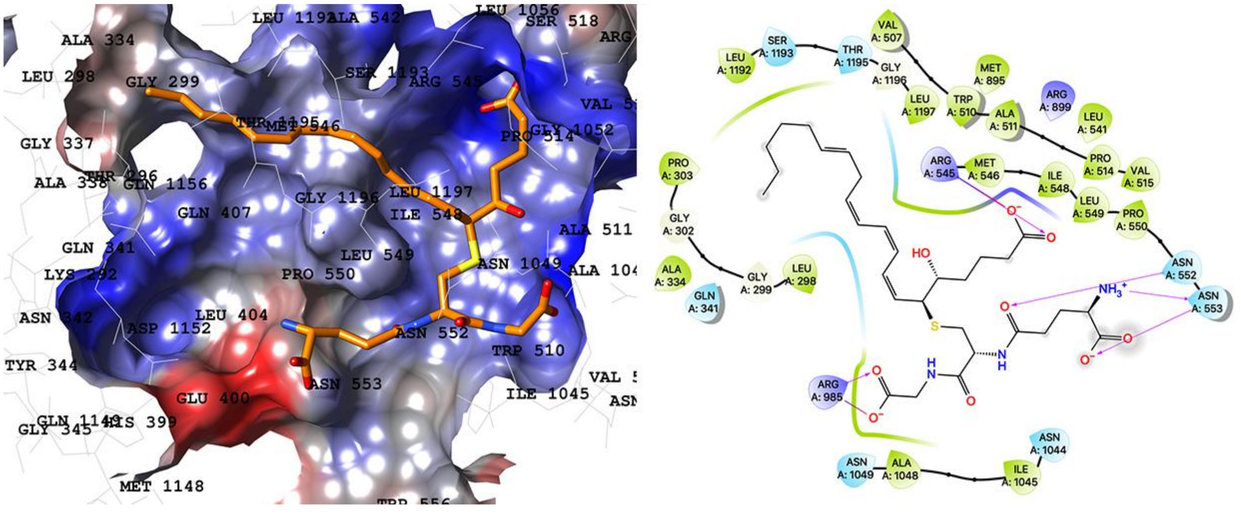

Fig. (S6). LTC_4-MRP7 complex.

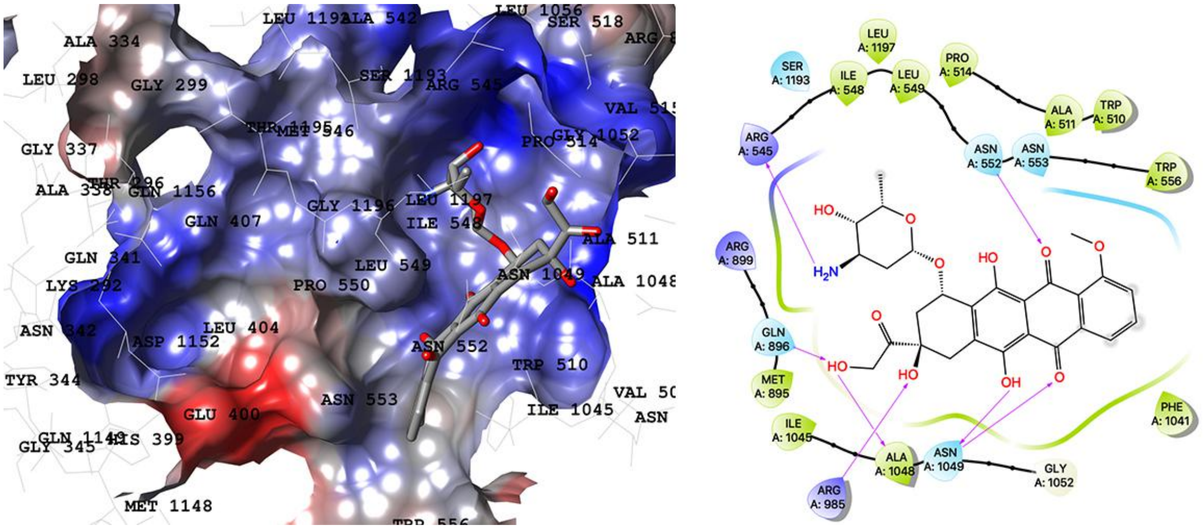

Fig. (S7). Doxorubicin-MRP7 complex.

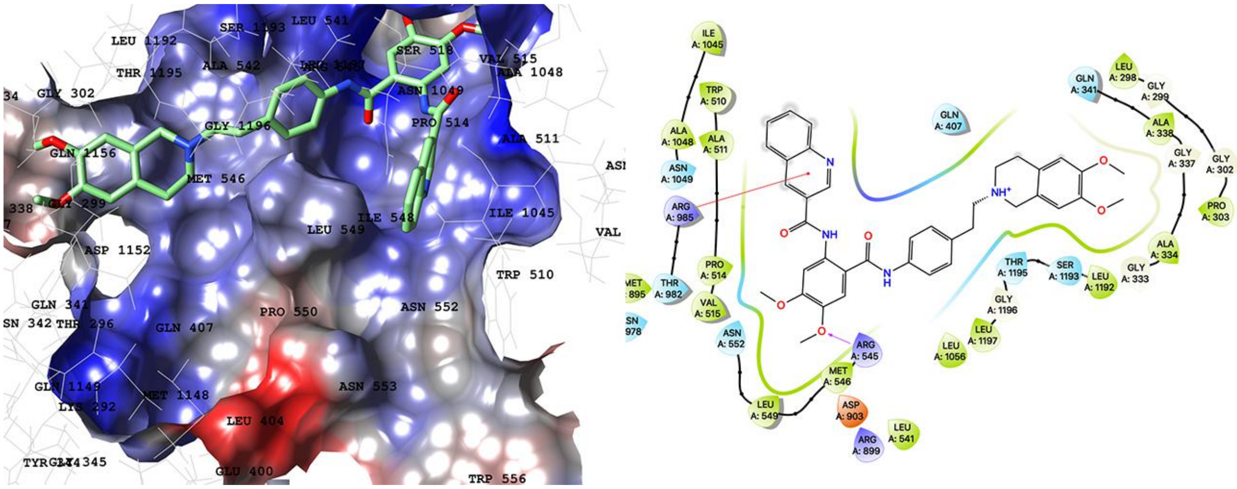

Fig. (S8). Tariquidar-MRP7 complex.

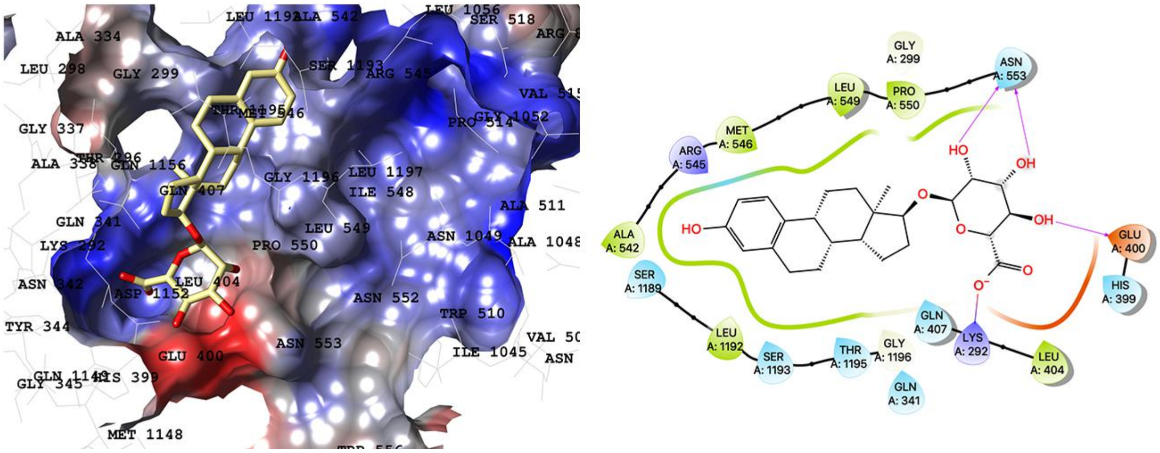

Fig. (S9). $E_217\beta G$-MRP7 complex.

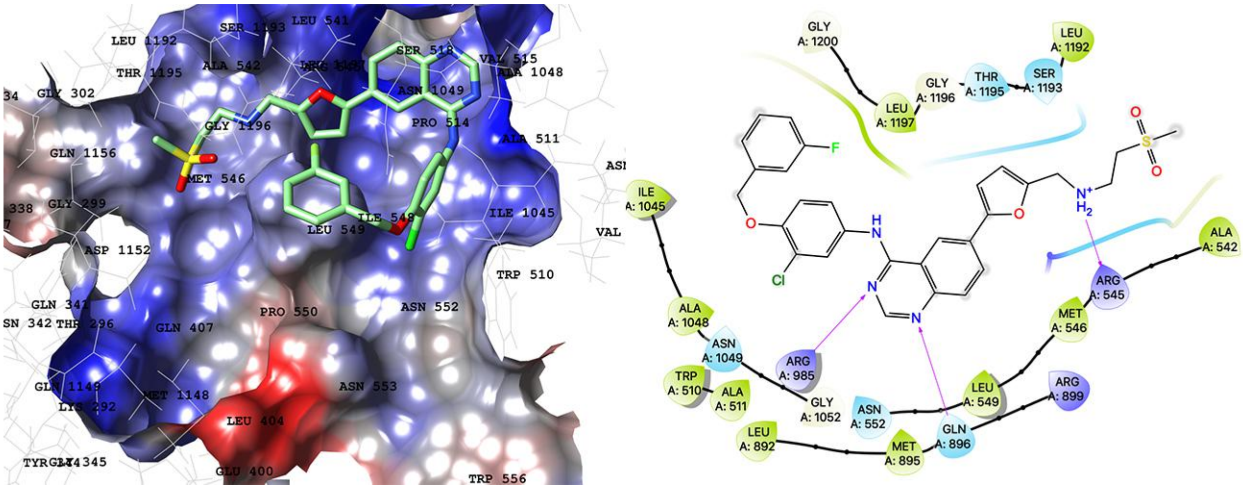

Fig. (S10). Lapatinib-MRP7 complex.

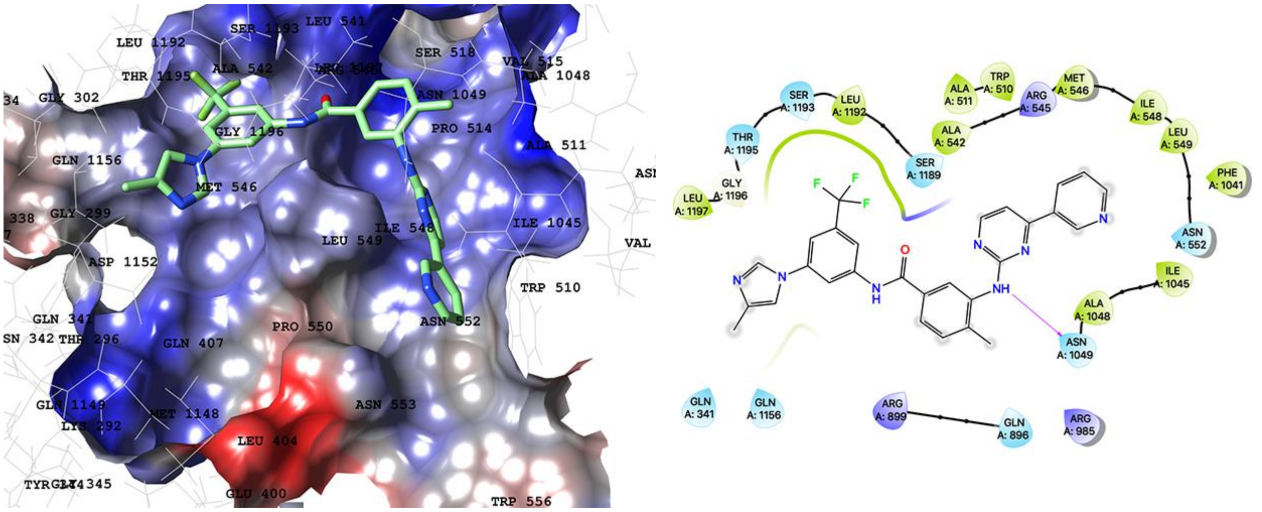

Fig. (S11). Nilotinib-MRP7 complex.

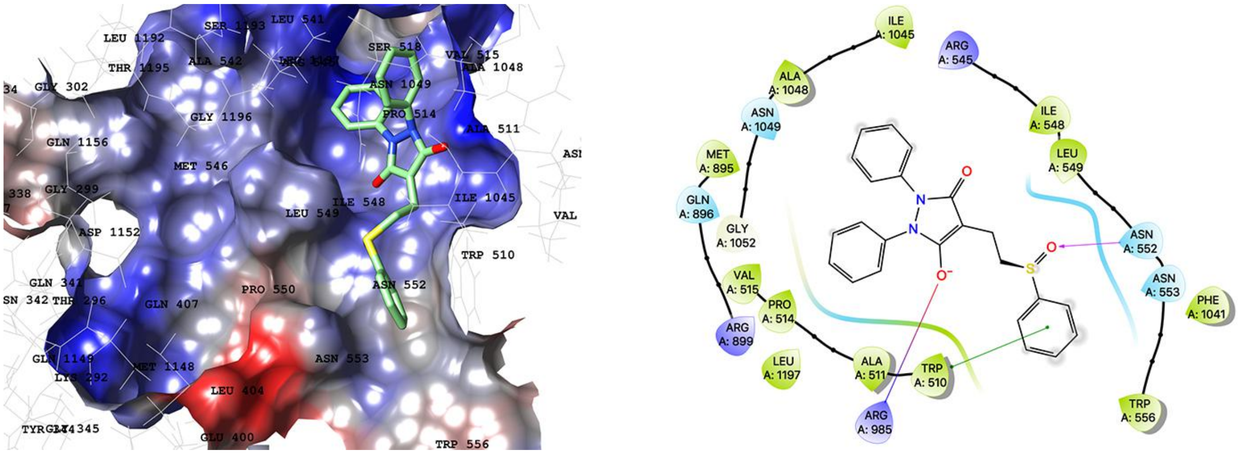

Fig. (S12). Sulfinpyrazone-MRP7 complex.

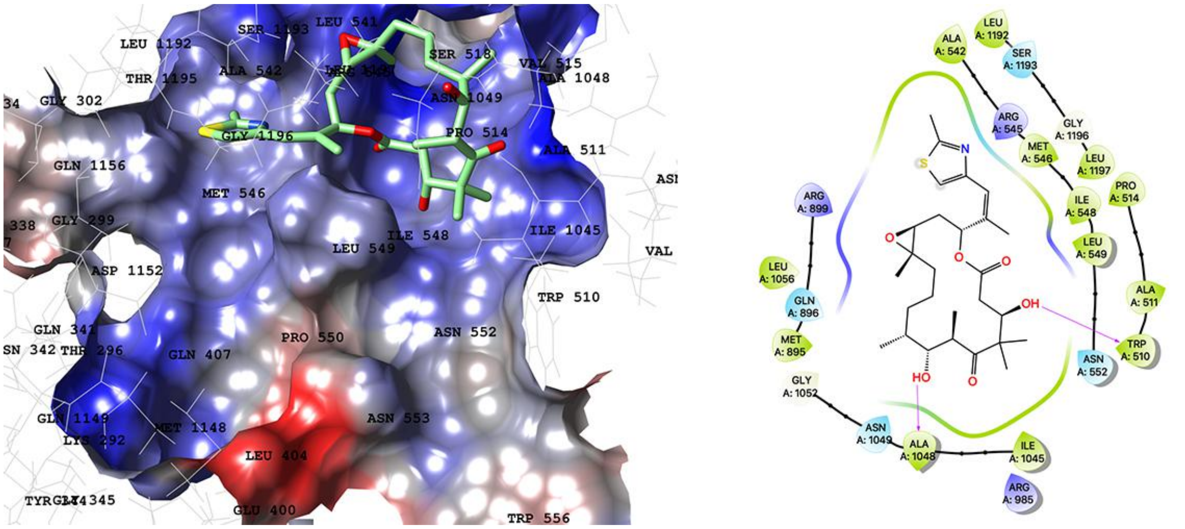

Fig. (S13). Epothilone B-MRP7 complex.

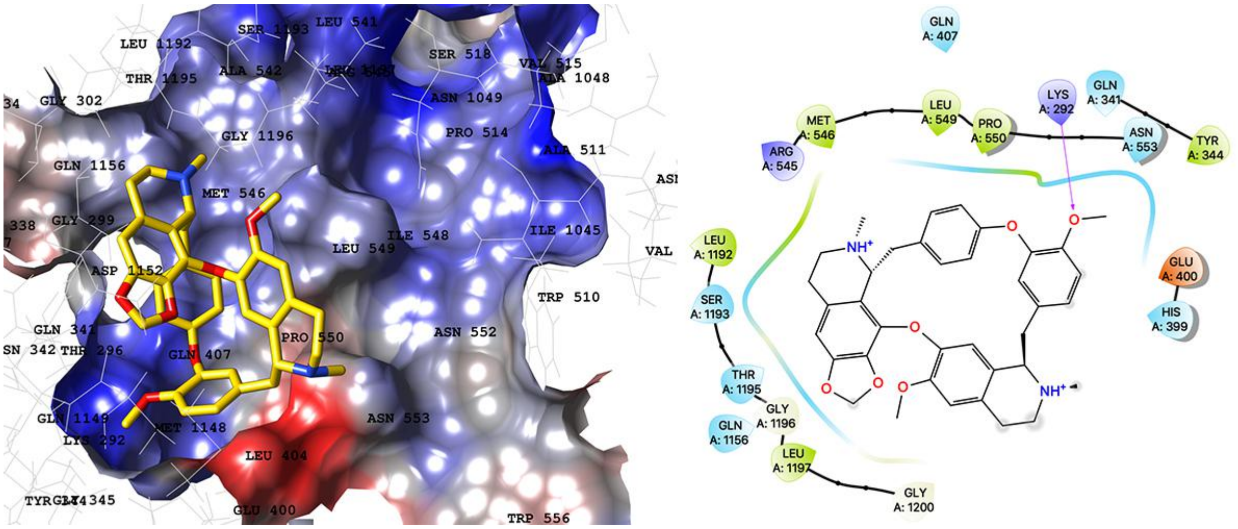

Fig. (S14). Cepharanthine-MRP7 complex.

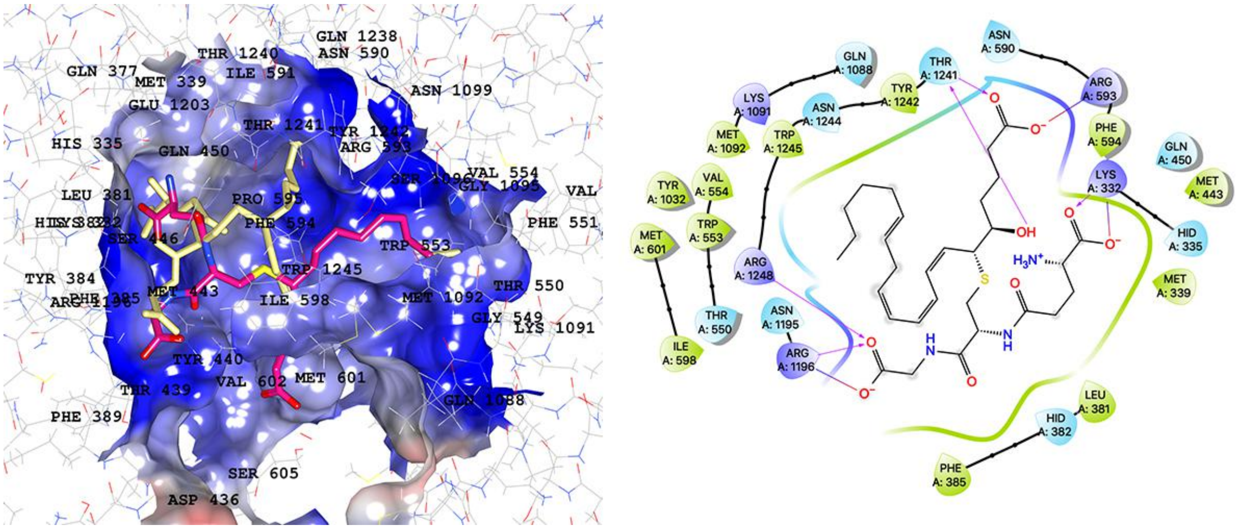

Fig. (S15). LTC_4-MRP1 complex. The docked pose was colored yellow. The co-crystalized LTC_4 in 5UJA was displayed magenta.

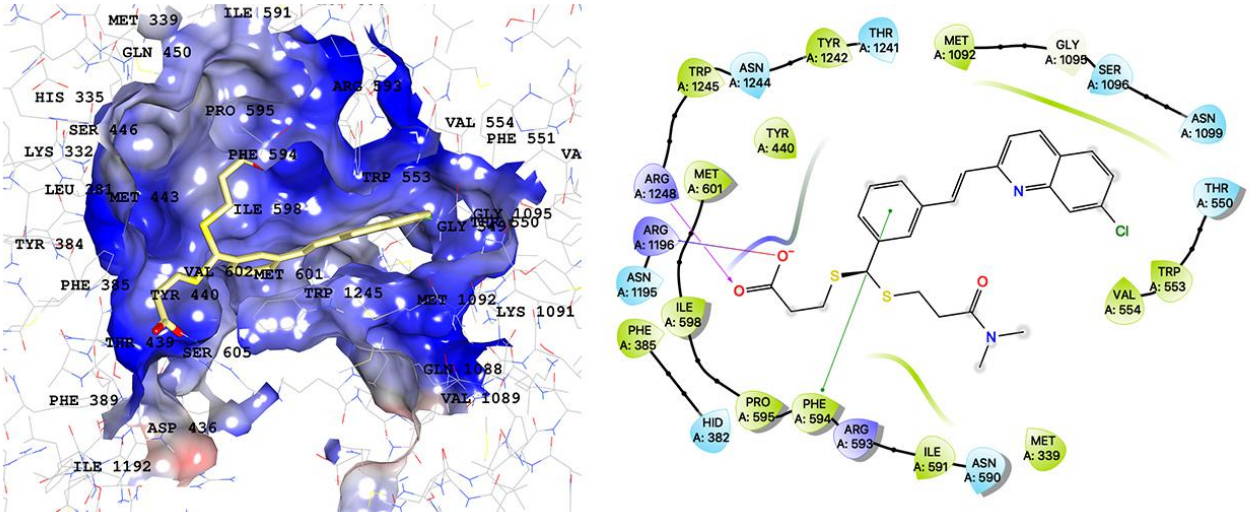

Fig. (S16). MK571-MRP1 complex.

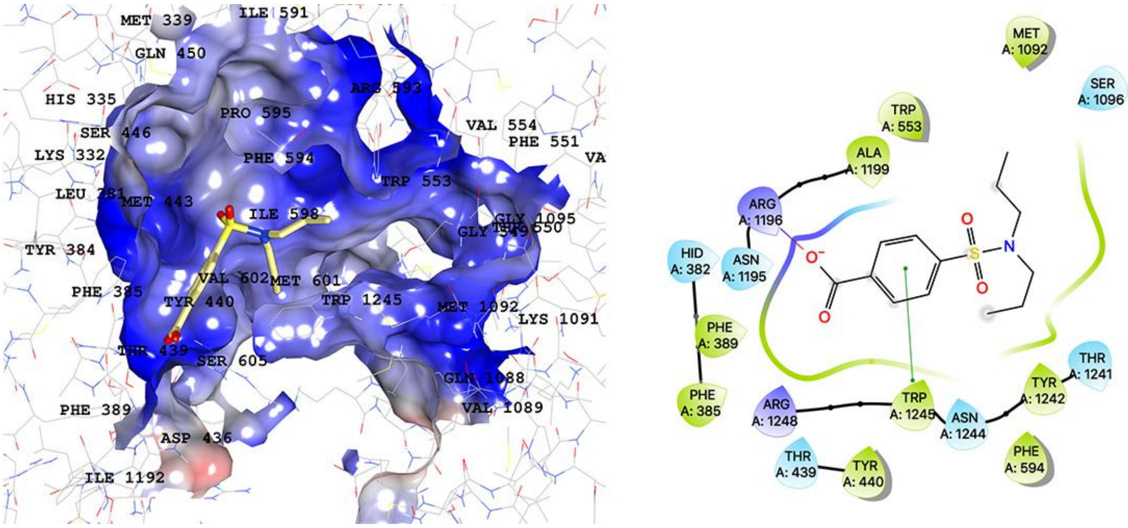

Fig. (S17). Probenecid-MRP1 complex.

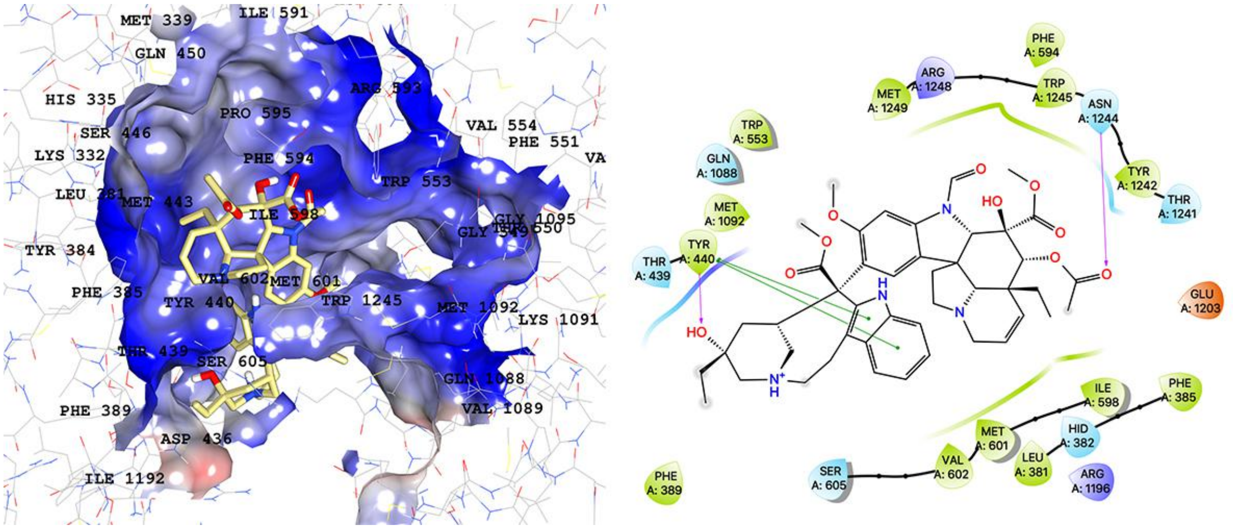

Fig. (S18). Vincristine-MRP1 complex.
